# Effects of Recreation on Animals Revealed as Widespread through a Global Systematic Review

**DOI:** 10.1371/journal.pone.0167259

**Published:** 2016-12-08

**Authors:** Courtney L. Larson, Sarah E. Reed, Adina M. Merenlender, Kevin R. Crooks

**Affiliations:** 1 Department of Fish, Wildlife, and Conservation Biology, Colorado State University, Fort Collins, Colorado, United States of America; 2 North America Program, Wildlife Conservation Society, Bozeman, Montana, United States of America; 3 Department of Environmental Science, Policy, and Management, University of California-Berkeley, Berkeley, California, United States of America; University of Hyogo, JAPAN

## Abstract

Outdoor recreation is typically assumed to be compatible with biodiversity conservation and is permitted in most protected areas worldwide. However, increasing numbers of studies are discovering negative effects of recreation on animals. We conducted a systematic review of the scientific literature and analyzed 274 articles on the effects of non-consumptive recreation on animals, across all geographic areas, taxonomic groups, and recreation activities. We quantified trends in publication rates and outlets, identified knowledge gaps, and assessed evidence for effects of recreation. Although publication rates are low and knowledge gaps remain, the evidence was clear with over 93% of reviewed articles documenting at least one effect of recreation on animals, the majority of which (59%) were classified as negative effects. Most articles focused on mammals (42% of articles) or birds (37%), locations in North America (37.7%) or Europe (26.6%), and individual-level responses (49%). Meanwhile, studies of amphibians, reptiles, and fish, locations in South America, Asia, and Africa, and responses at the population and community levels are lacking. Although responses are likely to be species-specific in many cases, some taxonomic groups (e.g., raptors, shorebirds, ungulates, and corals) had greater evidence for an effect of recreation. Counter to public perception, non-motorized activities had more evidence for a negative effect of recreation than motorized activities, with effects observed 1.2 times more frequently. Snow-based activities had more evidence for an effect than other types of recreation, with effects observed 1.3 times more frequently. Protecting biodiversity from potentially harmful effects of recreation is a primary concern for conservation planners and land managers who face increases in park visitation rates; accordingly, there is demand for science-based information to help solve these dilemmas.

## Introduction

Visitation to protected areas, ranging in scope from international ecotourism to local park visits, was recently estimated at 8 billion visits per year [1]. In the United States, the number of participants in outdoor recreation increased by 7.5% and total visitor days increased by 32.5% between 2000 and 2009 [2]. Driven in part by rapid growth in international tourism [3], recreation and ecotourism are also expanding in the developing world [4]; visits to protected areas in Africa, Asia, and Latin America increased by 2.5 to 5% between 1992 and 2006 [5].

Recreation is commonly assumed to be compatible with biodiversity conservation, in contrast to more well-known threats such as population growth and development at protected area edges [6,7] or subsistence use within reserves to help sustain local livelihoods [8]. Most protected areas have a dual mandate to conserve biodiversity and improve human welfare through resource use or outdoor recreation [8,9]. Accordingly, recreation is permitted in over 94% of International Union for Conservation of Nature (IUCN) protected areas globally (categories Ib-VI; [10,11]). In the United States and other developed nations, providing opportunities for outdoor recreation has historically been an important reason for the designation of protected areas [12], whereas in the developing world, ecotourism has been embraced as a potential win-win solution for poverty alleviation and conservation [8]. Furthermore, there are numerous benefits of outdoor recreation for human health and communities. People with access to natural areas have lower mortality rates [13], and outdoor play promotes mental and physical health in children [14]. Recreation and ecotourism can also be a source of economic revenue for protected areas and the communities around them [15,16], and can help garner support for conservation [17].

Despite these benefits, there is growing recognition that outdoor recreation can have negative impacts on biological communities. Recreation is a leading factor in endangerment of plant and animal species on United States federal lands [18], and is listed as a threat to 188 at-risk bird species globally [19]. Effects of recreation on animals include behavioral responses such as increased flight and vigilance [20,21]; changes in spatial or temporal habitat use [22,23]; declines in abundance, occupancy, or density [9,24,25]; physiological stress [26,27]; reduced reproductive success [28,29]; and altered species richness and community composition [30,31]. Many species respond similarly to human disturbance and predation risk, meaning that disturbance caused by recreation can force a trade-off between risk avoidance and fitness-enhancing activities such as foraging or caring for young [32].

Although there is a growing body of empirical studies of the effects of recreation on animals, a recent global review of the scientific literature does not exist. Early reviews [33–36] provide valuable definitions and conceptual frameworks, but were not systematic and need updating to reflect studies published in recent decades. In addition, contemporary reviews have restricted their scope by location or habitat type [37–39], taxonomic group [40–45], or recreation activity [46–48].

We conducted a global review of the published scientific literature to synthesize effects of non-consumptive recreation across all animal taxa. Such a review adds to the evidence base necessary to help bridge the gap between conservation science and practice [49]. To aid decision-makers faced with dilemmas about managing the demand for recreation while trying to fulfill mandates to protect species, it is critical to understand the degree to which biodiversity conservation and recreation are compatible, and under what circumstances. First, we examined trends in recreation research, including publication rates over time, geographic distribution, and study design. Second, we investigated which taxonomic groups were most commonly studied, and which had more or less evidence for effects of recreation. Similarly, we investigated which recreation activities and types of responses (e.g., behavioral, abundance, or survival) were most frequently measured, and what effects were observed. Finally, we examined management strategies proposed by the authors to avoid or mitigate these effects.

## Methods

### Search strategy

Because our objective was to locate studies of all animal species and all types of recreation, our search protocol was designed to produce a broad list of articles. We did not include taxonomic keywords since titles and abstracts often refer only to the study’s focal species. Instead, we limited the search to journals within four categories within the Institute for Scientific Information Web of Science database (Thompson Reuters, New York, NY, USA) that were the most relevant to our goals: biodiversity conservation, ecology, zoology, and behavioral sciences. From this list, we removed journals that were not published in English, or could not be reasonably expected to publish articles on recreation and animals (n = 166 journals included in the final list). We then searched the database with the Boolean search string: (ts = (touris* OR recreat*) AND so = (journal list)), where *ts* indicates topic keywords and *so* restricts the search to the list of 166 journals described above. This search strategy has high sensitivity (the proportion of all relevant information that the search locates) and low specificity (the proportion of search results that are relevant), which helps reduce bias and increase repeatability [50]. To reduce the effect of dissemination bias in our analysis, we included articles published in regional and lesser-known journals as well as the most widely-read publications [51]. Since our search strategy made use of the journal category feature within Web of Science, we were not able to replicate the search in other databases. However, our strategy produced a more thorough and comprehensive list of articles than if we had restricted our search with taxonomic keywords.

### Screening and data extraction

**Fig 1:**
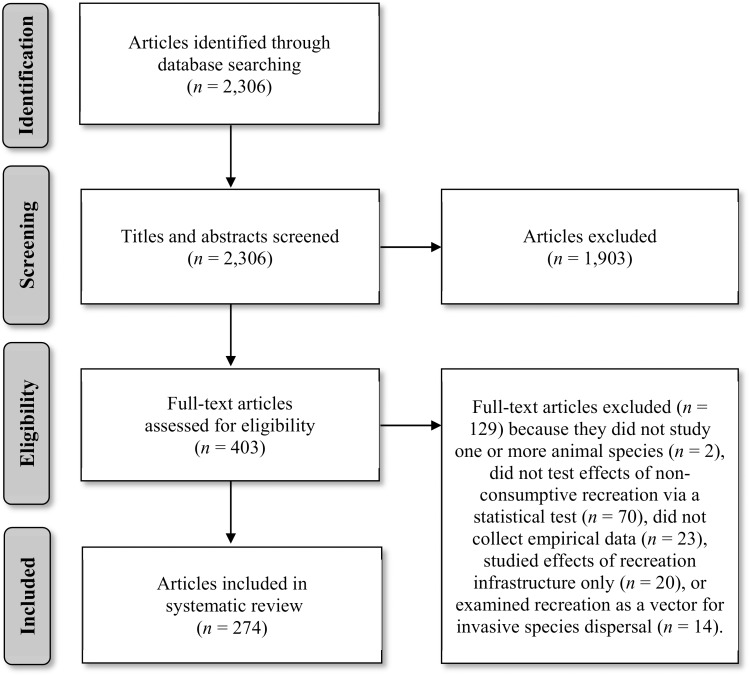
PRISMA literature search flow diagram. The number of studies that were located, retained, and discarded are shown at each stage of the literature review process.

Our keyword search (performed 30 January 2013 and again on 21 March 2016) resulted in a comprehensive list of 2,306 articles. We first reviewed titles and abstracts and eliminated obviously irrelevant records (e.g., tourism management papers with no wildlife component; Fig 1). We then reviewed the full text of the remaining 403 articles and assessed them against our inclusion criteria, recording the reason for rejection if necessary [50]. We excluded consumptive activities, which we define following Duffus and Dearden [34] as activities that “purposefully remove or permanently affect wildlife” (e.g., hunting, fishing). We focused on non-consumptive forms of recreation (e.g., hiking, skiing) because these activities are permitted more widely throughout protected areas. However, studies examining consumptive activities as a source of disturbance for non-target species (e.g., effects of fishing on waterbirds; [52]) were retained. We also rejected articles if they did not study one or more animal species (*n =* 2), did not test effects of non-consumptive recreation via a statistical test (*n =* 70), did not collect empirical field data (e.g., were review or simulation articles; *n =* 23), studied the effects of recreation infrastructure independently of human activity (e.g., presence of ski lifts; *n =* 20), or examined recreation as a vector for invasive species dispersal (*n =* 14). Experimental treatments designed to mimic recreational activities were included. The final list included 274 articles (S1 Appendix) with 2,048 distinct results.

Data collected from each article included publication information, geographic location (country and continent), study design, taxonomic group(s), recreation activities, response types and effects found, and management recommendations (Table 1). For articles that studied multiple species, recreation activities, or response types, we treated each combination of variables as a separate “result,” rather than attempting to determine an overall effect for each article, which would ignore valuable findings from within each article. For example, Banks and Bryant [24] examined the effects of hiking and dog-walking on bird abundance and richness, so we recorded four combinations of “results” in our database. While results from the same study often rely on the same animal populations, locations, and data collection efforts, we examined each result separately since effects often differed. Because each article could be considered an experimental unit, we added a random effect for article in the analysis to control for this potentially confounding factor (see “Statistical analysis”).

**Table 1:** List of variables collected from articles included in the review of the effects of non-consumptive recreation on animals.

Category	Variable	Description or list of categories	Data type
Publication	Author(s)		text
	Title		text
	Journal		text
	Journal type	Behavior, conservation, ecology, ecosystem/region-specific, general biology, taxa-specific, zoology/wildlife, other	categorical
	Publication year		numeric
Geographic	Continent		categorical
	Country		text
	Habitat type	Agricultural, beach, desert, forest, freshwater, grassland, marine, polar, shoreline, urban, scrub/shrub, tundra, wetland, other	categorical
Study design	Measure of recreation*	Direct observation, experimental treatment, expert opinion, remote monitoring, permitted use, proxy	categorical
	Experiment	Was it an experimental study?	yes/no
	Control	Did the study include a control treatment? (e.g. a “no-recreation” site)	yes/no
	Replication	Did the study replicate treatments, study sites, observation periods, etc?	yes/no
Effect	Effect*	Did the authors find a significant recreation impact?	yes/no
	Effect direction*	Positive, negative, unclear	categorical
Taxonomic	Multiple species	Were multiple species studied?	yes/no
	Taxa group	Amphibian, bird, fish, invertebrate, mammal, reptile	categorical
	Scientific name*		text
	Common name*		text
Recreation	Activity*	Alpine skiing, beach use, biking, boating (non-motorized), camping, nordic ski/snowshoeing, dog-walking, equestrian, hiking/running, motorized (boat), motorized (land), motorized (snow), swimming/diving, wildlife feeding, wildlife viewing (boat), wildlife viewing (land), other (aquatic), other (terrestrial)	categorical
Response	Type*	Abundance, behavioral, community (species richness, diversity, or composition), occurrence, physiological, reproductive, survival, other	categorical
Management	Recommendations	Cap visitation, improve infrastructure, rule change, staff training, spatial restrictions, temporal restrictions, visitor education, none, other	categorical

* For articles that studied multiple species, recreation activities, or response variables, we treated each combination of variables as a separate “result,” and recorded the information marked with an asterisk (*) for each result individually.

The “effect” variable (Table 1), which was the response variable for several of our research questions, was a binary variable indicating whether the recreation effect documented by the authors was statistically significant (as defined by the authors). We categorized all significant effects as negative, positive, or unclear. Negative responses were consistent with the following effects of recreational disturbance at the community, population, or individual (behavioral or physiological) levels: decreased species richness or diversity; decreased survival, reproduction, occurrence, or abundance; behaviors typically assumed to reflect negative responses to anthropogenic disturbance (e.g., decreased foraging or increased vigilance); and physiological condition typically assumed to reflect disturbance effects (e.g., decreased weight or increased stress). Conversely, positive responses were in the opposite direction. We were unable to classify some responses as positive or negative and labeled them “unclear.” Examples of unclear effects were behavioral responses that did not have obvious fitness consequences (e.g., decreased vocalizing) and results with non-linear responses (e.g., highest reproductive success at an intermediate level of recreation). We note that positive responses do not necessarily imply beneficial outcomes for biodiversity conservation; for example, an increase in species richness could be attributable to an increase in non-native species.

We caution that a statistically significant effect of recreation does not necessarily provide insight into the effect’s magnitude or biological significance. Authors may also include statistically significant results while omitting non-significant findings due to publication bias [53]. A formal meta-analysis framework can help researchers summarize effect sizes and detect and adjust for publication bias [54], but the study design must be similar across all studies included, with comparable predictor and response variables [55]. This was not feasible given the broad scope of our review, and accordingly, we do not make statistical comparisons among groups. Ultimately, we believe our approach provides a meaningful representation of the weight of evidence that currently exists.

### Publication trends and geographic distribution

We summarized the number of articles by publication year, journal type, country, continent, and habitat type. Journals were classified into eight broad types using the journal title and online aims and scope statement to identify the appropriate primary category. Articles were also assigned to one or more habitat classes on the basis of authors’ descriptions (Table 1).

### Study design

To examine how recreation studies have been designed and conducted, we recorded the proportion of articles that used an experimental design and included controls and replication. For our purposes, any kind of an experimental treatment (e.g., experimental boat passes near a raptor nest; [56]) counted as an experimental design, and any treatment or site without recreation counted as a control. We also examined the method used to measure recreation: direct observation (with human observers), experimental treatment (e.g., researchers simulating recreation activities), expert opinion, remote monitoring (e.g., automatic counters), permitted use (e.g., whether a site was open to a specific recreational activity), or proxy variables (e.g., car counts).

### Taxonomic groups

We examined differences in research focus and evidence for recreation effects among six broad taxonomic groups: amphibians, birds, fish, invertebrates, mammals, and reptiles. We divided groups with sufficient sample size (≥ 15 results on ≥ 3 different species) into narrower taxonomic classifications (Classes for invertebrates and fish; Orders for birds, mammals, and reptiles; amphibians were omitted due to small sample size). We then subdivided Classes or Orders with sufficient sample sizes (≥ 15 results on ≥ 3 different species) once again into Orders or Families. We also grouped species by their IUCN status [57].

### Recreation activities

We grouped recreation activities into 18 types (Table 1) and created broader categories for more general comparisons: winter terrestrial (snow and ice-based activities such as skiing and snowmobiling), summer terrestrial (land activities not requiring snow or ice), and aquatic activities. We also compared motorized and non-motorized activities.

### Response types

We categorized animal responses into eight types: community (species richness, diversity, or composition metrics), survival, reproduction, abundance, occurrence, behavior, and physiological measures, as well as “other” responses (e.g., sex ratio). For more general comparisons, we also grouped the response types hierarchically into community-, population- (survival, reproduction, abundance, and occurrence), and individual-level (behavior and physiological) responses.

### Management recommendations

To qualify the management recommendations noted in the articles and provide a useful synthesis for land managers, we categorized recommended management actions as follows: spatial restrictions, capping visitation, increasing visitor education, temporal restrictions, improving infrastructure, adding or changing rules, enforcement of existing rules, staff training, or “other” (Table 2). Calls for additional research, although common in the literature, were not considered to be management recommendations.

**Table 2:** General management recommendations suggested by authors of articles included in the review.

Recommendation	Examples	Frequency (%)*
Spatial restrictions	Designate a trail-free area within protected area; establish minimum approach distances to animals	32.1
Visitor education	Educate SCUBA divers about the impacts of human contact on coral; instruct visitors about effects of noise on sensitive species	15.0
Cap visitation	Limit the number of visitors that can enter the area per day	14.2
Temporal restrictions	Limit recreational access during the breeding season	13.1
Rule change	Restrict boat speed in sensitive areas; prohibit wildlife feeding	9.9
Physical improvements	Restore habitat; install fencing around sensitive areas	9.5
Other	Species translocations; increased use of private land for conservation	8.8
Enforcement	Enforce leash laws; keep people on trails	6.9
Staff training	Train staff to recognize signs of animal disturbance	2.2
No recommendations		40.5

* Percentages do not sum to 100 because some articles made more than one management recommendation.

### Statistical analysis

We used linear regression to assess trends in the total number of articles over time as well as the proportion of included articles out of the total publication volume in the selected journals. To assess gaps in the literature, we used chi-square goodness of fit tests to determine if the distribution of articles differed significantly from an expected distribution. For journal type, the expected distribution was the proportion of journals in the journal set that belonged to each type. For geographic distribution, we compared the distribution of articles by continent to the total land area and human population density of each continent. For IUCN status and taxonomic groups, the expected distribution was the number of known species in each group, starting with the broadest groups and progressing down to Family when possible [57]. We did not use chi-square tests if articles were counted under more than one category (e.g., articles examining multiple types of recreation, such as hiking, biking, and equestrian) since this violates the assumption of independence.

We estimated the amount of evidence for a recreation impact as the overall percentage of results that found a statistically significant effect of recreation. These percentages were estimated for results summarized by taxonomic groups, recreation activities, and response types. Because most articles included multiple results, the percentages (± SE) we report are least-squares means and standard errors obtained from models that included article as a random effect. We used generalized linear mixed models (GLMMs) with a logit link function to estimate the frequency of overall effects among taxonomic groups, recreation activities, and response types, and we used proportional odds models [58] to estimate the proportion of overall effects that were negative, positive, or unclear. All statistical analyses were conducted in R using packages lme4, ordinal, and lsmeans [59–62].

## Results

### Publication trends and geographic distribution

**Fig 2:**
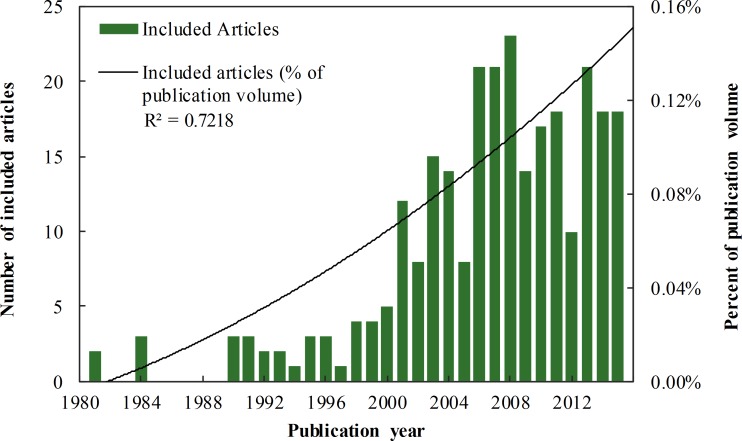
Published articles on the effects of non-consumptive recreation on animals by publication year. The numbers of articles are shown as raw numbers (shaded bars) and as percentages of the overall publication volume in the journal set used in this review (trendline; a second order polynomial function).

The earliest articles discovered by our search were published in 1981, and the peak year was 2008 with 23 articles. The number of articles published per year that met our criteria increased 23.5% on average per year from 1981 to 2015 (β = 0.66, 95% CI = (0.53, 0.80), *p* < 0.0001). This increase was not solely a result of increasing publication volume; the proportion of included articles out of the total articles published in the journal set increased by 8.8% on average per year (β = 0.000043, 95% CI = (0.000033, 0.000053), *p* < 0.0001; Fig 2). The distributions of the journal set into journal types (e.g., conservation, wildlife) and individual articles into journal types were significantly different (χ^2^ = 632.4, df = 7, *p* < 0.0001). Most of the included articles were published in conservation (38.7%) and wildlife (19.7%) journals, followed by ecology (13.5%), taxa-specific (13.1%), ecosystem or region-specific (9.9%), and behavior journals (3.3%); very few articles were published in general biology (0.7%) or other (0.7%) journal categories.

**Fig 3:**
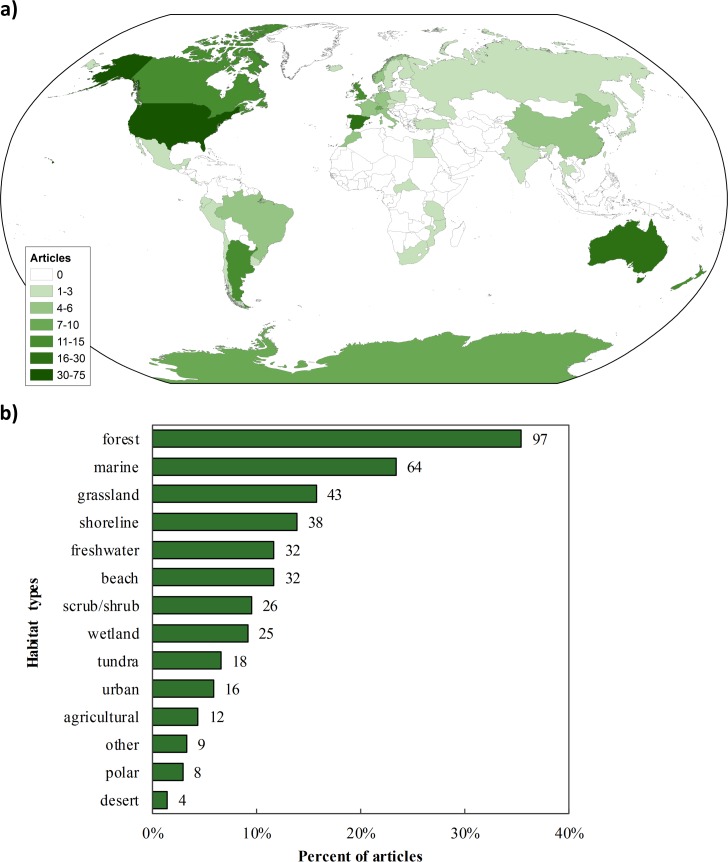
Distribution of published articles on the effects of non-consumptive recreation on animal species. Panel (a) shows the countries where studies were conducted, and panel b) shows the distribution of studies into major habitat type(s). Since some studies involved multiple habitat types, the sum (424) is greater than the total number of articles (274). Numbers at the end of bars represent the total number of articles in each category.

Geographically, studies of recreation on animals were conducted mostly in North America (37.7%), Europe (26.6%), and Oceania (13.1%), and relatively few in South America (9.1%), Asia (5.5%), Africa (5.1%), and Antarctica (2.9%; Fig 3A). This distribution among continents was not proportional to the land area (χ^2^ = 366.3, df = 6, *p* < 0.0001) nor human population density (χ^2^ > 500, df = 6, *p* < 0.0001) of the continents. The United States accounted for 27.0% of the articles, followed by Australia (7.7%), Spain (5.8%), New Zealand (5.5%), the United Kingdom (4.7%), Argentina (4.4%), and Canada (4.4%). Most studies were conducted in forest (35.4%), marine (23.4%), grassland (15.7%), and shoreline (13.9%) habitats (Fig 3B). The least well-studied habitat types were polar (2.9%), and desert (1.5%), as well as human-modified habitats (agricultural and urban, representing 10.2% of articles combined).

### Study design

Less than one-third (30.3%) of the articles contained an experimental component, and 60.9% of articles contained controls. Most (85.4%) articles had replication of study sites, treatments, or groups. Direct observation was the most common method for measuring recreation (38.1% of results), followed by proxy variables (19.9%), expert opinion (19.6%), and experimental treatment (18.0%). Permitted use as a measure of recreation was less common (12.5%), as was remote monitoring (6.7%).

### Taxonomic groups

**Fig 4:**
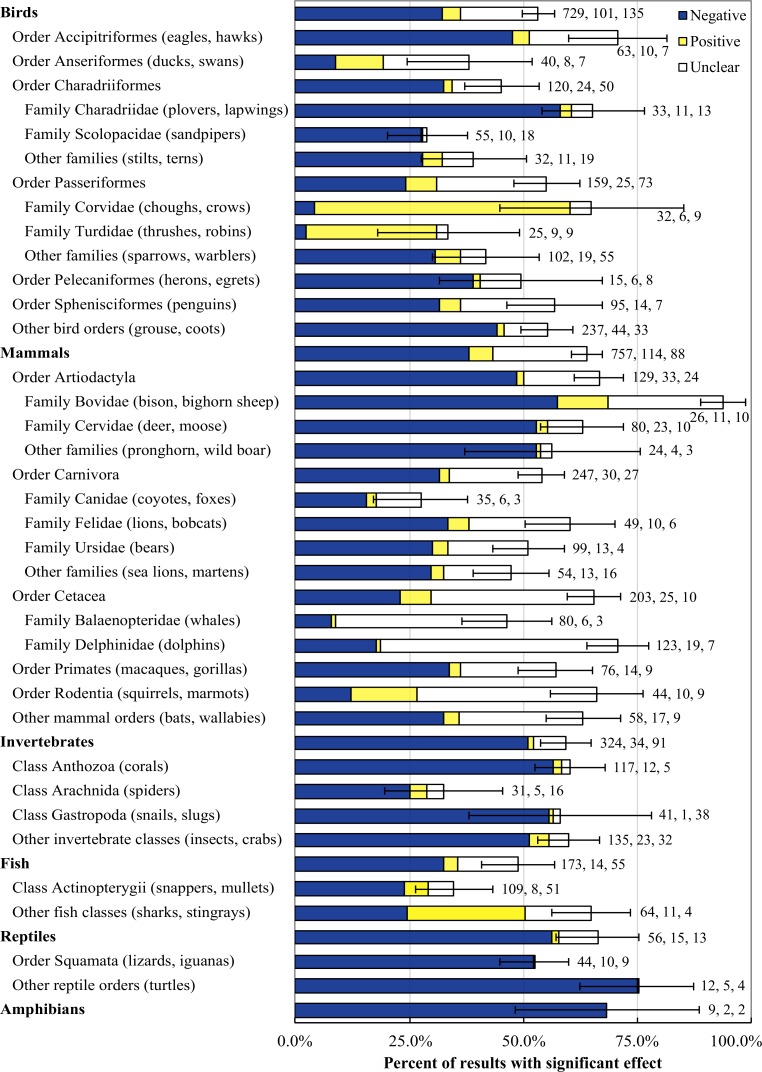
Evidence for an effect of recreation by taxonomic group. Evidence is measured as the proportion of results that were statistically significant. For articles that studied multiple recreation activities, species, or response variables, each combination of variables was treated as a separate result. Common names are examples of species occurring in the included articles. We present taxonomic groups that have at least 15 results and 5 species represented; the remaining taxa are included in “other” categories for comparative purposes. Numbers following bars show the number of results, number of articles, and count of unique species. Articles that studied functional groups or communities rather than individual species (e.g., insectivorous birds) were added to the relevant “other” category and were not counted as species. Error bars show standard error for the sum of all effects.

Research effort in our sample of articles was not proportional to the number of species within all taxonomic groups at the broadest level (χ^2^ = 377.3, df = 5, *p* < 0.0001), nor to the number of species in bird (χ^2^ = 988.7, df = 5, *p* < 0.0001) and mammal (χ^2^ = 290.3, df = 3, *p* < 0.0001) Orders or invertebrate Classes (χ^2^ = 98.1, df = 2, *p* < 0.0001; Fig 4). Mammals (41.6%) and birds (36.9% of articles) were the focus of the majority of recreation studies, followed by invertebrates (12.4%), reptiles (5.5%), fish (5.1%), and amphibians (0.7%). Studies of a single species were more common (69.0%) than those that examined at least two species. Research on mammals focused mainly on ungulates (28.9%), carnivores (26.3% of articles), cetaceans (21.9%), and primates (12.3%). Among birds, the most commonly researched Orders were Passeriformes (passerine birds; 24.8% of articles), Charadriiformes (wading birds and gulls; 23.8%), Sphenisciformes (penguins; 13.9%), and Accipitriformes (hawks, eagles, vultures; 9.9%). Many of the invertebrate studies (35.2%) focused on the effects of snorkeling or SCUBA diving on corals, followed by studies on arachnids, bivalves, and insects (each 14.7%). The most commonly studied fish Class was Actinopterygii (ray-finned fish; 57.1%), followed by Chondrichthyes (sharks, stingrays; 42.9%). Research on reptiles focused on Orders Squamata (lizards, snakes; 78.6%) and Testudines (turtles; 21.4%).

We identified the IUCN status of the species for 68.7% of results, representing 305 unique species; the remaining results examined multiple species or species not evaluated by the IUCN. The distribution of these results into IUCN status categories was not proportional to the distribution of all animal species into these categories (χ^2^ = 108.3, df = 5, *p* < 0.0001), with many more species than expected in the least concern category (80.7%), slightly more than expected in the near threatened (6.9%), and fewer than expected in the data deficient (1.6%), vulnerable (6.5%), endangered (3.6%), and critically endangered (0.1%) categories. Endangered species that were studied included three mammals (black howler monkey *Alouatta pigra*, Hector’s dolphin *Cephalorhynchus hectori*, and the Barbary macaque *Macaca sylvanus*), three fish (dusky grouper *Epinephelus marginatus*, Nassau grouper *Epinephelus striatus*, and the brownstriped gaunt *Anisotremus moricandi*), two birds (Egyptian vulture *Neophron percnopterus* and the yellow-eyed penguin *Megatypes antipodes*), two reptiles (wood turtle *Glyptemys insculpta* and Lilford’s wall lizard *Podarcis lilfordi*), and the boulder star coral *Montastraea annularis*. The only critically endangered animals were the Western lowland gorilla *Gorilla gorilla gorilla* and the Mexican howler monkey *Alouatta palliata mexicana*.

Of the 274 articles analyzed, 93.1% documented at least one effect of recreation on animal populations, individuals, or communities. Negative effects of recreation were the most frequent (59.4%), followed by unclear (25.9%) and positive (14.7%) effects. Most (83.6%) of the unclear effects were behavioral responses.

Taxonomic groups with the most negative effects were amphibians (68.4 ± 20.2% of results), reptiles (56.3 ± 9.2%), and invertebrates (51.0 ± 5.1%), while mammals (5.3 ± 1.9%) and birds (4.3 ± 2.0%) had the most positive effects (Fig 4). Among bird Orders, evidence for overall and negative effects was greatest in Accipitriformes (e.g., eagles, hawks; 70.7 ± 10.7 and 47.7 ± 24.4%; Fig 4). Positive effects were greatest in Anseriformes (e.g., ducks, swans; 10.4 ± 22.6%) and Passeriformes (passerine birds; 6.9 ± 7.7%). Evidence of negative effects among Charadriiformes Families was greatest in Charadriidae (e.g., plovers, lapwings; 58.2 ± 18.6%). Among Passeriformes Families, Corvidae (e.g., crows, choughs) had the most positive effects (56.0 ± 4.9%). Among mammal Orders, Artiodactyla (even-toed ungulates) had the most negative effects (48.5 ± 8.0%) and Rodentia (rodents) had the most positive effects (14.4 ± 12.3%). At the family level, Bovidae (e.g., bison, bighorn sheep) had by far the most overall effects (93.8 ± 19.3%) and Delphinidae (dolphins) was also high (70.8 ± 6.8%). Several invertebrate Classes had considerable negative effects, including Anthozoa (corals; 56.6 ± 4.2%), Gastropoda (e.g., snails, slugs; 55.5 ± 6.7%), and “other” (e.g., insects, crabs; 51.4% ± 6.0%). Finally, the “other” grouping of fish Classes (e.g., sharks, stingrays) had more evidence for an overall and positive effect (64.9 ± 8.7% overall and 25.8 ± 15.7% positive) than Actinopterygii (ray-finned fish; 34.8 ± 8.5% overall and 5.4 ± 9.2% positive). Of the reptile orders, Order Testudines (turtles) had more overall effects (75.0 ± 12.5%) effects than Order Squamata (lizards, iguanas; 52.3 ± 7.5). For both Orders, all of the effects were negative. Low sample sizes precluded comparisons among amphibian taxa.

### Recreation activities

**Fig 5:**
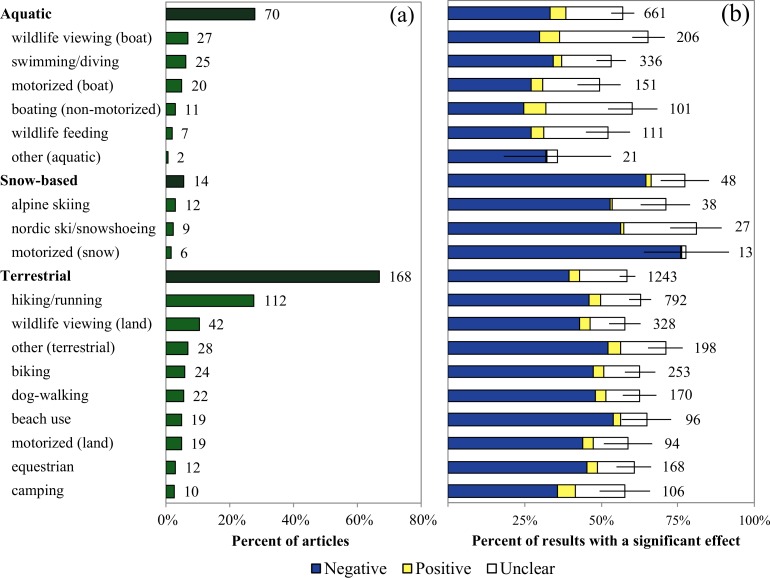
Recreation activities in the articles included in this review. Panel (a) shows the percent of articles that included each recreation activity (numbers of articles follow the bars), and panel (b) shows the percent of results in which a statistically significant effect of recreation on an animal species was observed (number of results follow the bars). Total percentages are divided into negative, positive, and unclear effects of recreation. Error bars show standard error for the sum of all effects.

The articles in our sample examined a wide variety of recreation activities (Fig 5A). Summer terrestrial activities were the most common, studied by 66.7% of articles, followed by aquatic (27.8%) and winter terrestrial (5.6%). Motorized forms of recreation, including off-highway vehicles, snowmobiles, and motorized boats, were examined in 26.3% of articles. Hiking was studied much more often than any other recreation activity (27.5% of articles). Wildlife viewing was also relatively frequently studied, with 10.3% of articles studying land-based and 6.6% studying boat-based wildlife viewing.

Winter terrestrial activities had the most evidence of overall (77.3 ± 7.8% of results; Fig 5B) and negative (64.4 ± 10.1%) effects, compared to 58.5 ± 2.7% (overall) and 39.6 ± 4.6% (negative) for other terrestrial and 57.0 ± 3.8% (overall) and 33.4 ± 7.1% (negative) for aquatic activities. Although motorized and non-motorized activities had similar evidence for overall effects (57.0 ± 5.1% and 58.4 ± 2.5%), non-motorized had greater negative effects (40.3 ± 4.0% versus 34.0 ± 8.6%). Activities with the most evidence of overall effects included each of the snow activities (cross-country ski/snowshoeing: 81.0 ± 8.6%, motorized–snow: 77.8 ± 13.9%, alpine skiing: 71.0 ± 8.2%), as well as boat-based wildlife viewing (65.4 ± 5.4%) and beach use (64.8 ± 8.2%; Fig 5B).

### Response types

**Fig 6:**
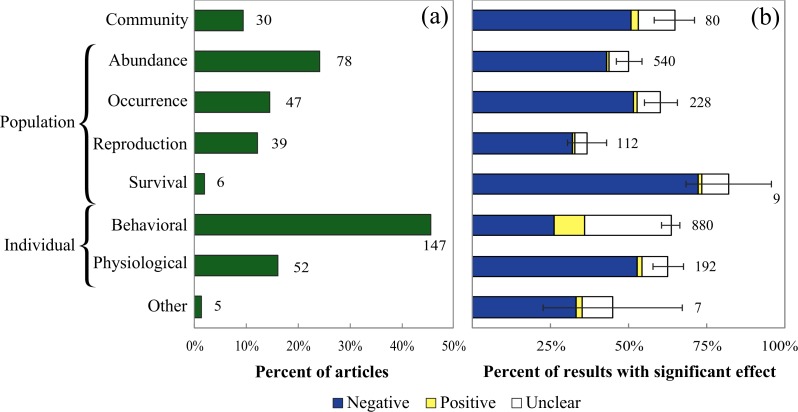
Types of animal responses to recreation in the articles included in this review. Response types have been categorized into community-, population-, and individual-level responses. Panel a) shows the percent of articles in which each response type is tested (numbers of articles follow the bars). Panel b) shows the percent of results in which a statistically significant effect of recreation on an animal species was observed (number of results follow the bars). Total percentages are divided into negative, positive, and unclear effects of recreation. Error bars show standard error for the sum of all effects.

Response types were not studied evenly; behavioral (45.5% of articles) and abundance (24.1%) responses to recreation were the most common (Fig 6A). Only 9.3% of articles measured community metrics (species richness, diversity, or composition) and 1.9% measured survival. Omitting survival responses due to small sample size, community responses had the most overall effects (64.6 ± 6.6% of results), followed by behavioral (63.5 ± 2.8%) and physiological (62.5 ± 4.9%) responses; reproductive responses (36.7 ± 6.3%) had the fewest overall effects (Fig 6B). Physiological (52.7 ± 4.8%) and occurrence (51.3 ± 4.6%) responses had the most negative effects, while behavioral responses had the most positive effects (9.8 ± 2.5%).

### Management recommendations

More than one-third (40.5%) of the included articles did not provide management recommendations (Table 2). Of those that did include recommendations, the most common types were spatial restrictions (32.1%), visitor education (15.0%), and limiting visitation (14.2%), Enforcement of existing rules (6.9%) and staff training (2.2%) were the least frequently suggested management categories.

## Discussion

Although published research on recreation effects on animals increased by an order of magnitude from 1981 to 2015, the percentage of the literature devoted to the subject remains small (0.16% of publication volume of the target journals in the peak year), and many gaps in knowledge remain. The literature is geographically biased in favor of North America and Europe, and taxonomically biased toward birds and mammals. Over 93% of reviewed articles documented at least one effect of recreation, and as expected, the majority of these effects were negative. Non-motorized and winter terrestrial activities had notable evidence for negative effects. Additionally, some of the least studied taxonomic groups (reptiles, amphibians, and invertebrates) had the greatest evidence for negative effects of recreation.

Though the amount of literature on this topic has increased in recent decades, it may not be reaching a broad audience even among conservation scientists and wildlife ecologists. Over 20% of articles were published in journals specific to a taxonomic group, geographic region, or ecosystem, whereas few were published in the broadest journals. Since the broadest journals are also among the highest-impact publications (e.g., *Science*, *Nature*), this could also indicate that the topic of recreation impacts on animals is not viewed as important within the peer-reviewed literature.

The articles had a strong geographic bias toward North America and Europe. This reflects global patterns in visitation to protected areas since over 80% of visits occur in these two continents [1]. A surprising number of studies were conducted in Antarctica, as a result of a growing ecotourism industry that often includes visits to penguin colonies [63]. As South America, Africa, and Asia contain most of the world’s biodiversity hotspots [64] as well as popular ecotourism destinations including Brazil, South Africa, Thailand, and Indonesia [65], we see an immediate need for studies of recreation effects in these areas. The few studies conducted in tundra, polar, and desert habitat types is likely a result of low rates of recreation and tourism occurring in these areas. However, our findings and those of Sato et al. [39] about the impacts of alpine activities indicate that it is an important area for future study.

Further, the distribution of articles among broad taxonomic groups was skewed in favor of mammals and birds, a trend consistent with conservation science as a whole [66]. However, these are large, diverse groups that still warrant more research; for example, passerine birds were the most frequently studied avian Order in our set of articles, but the 73 species examined therein comprise ~1% of the 5,000+ species in the Order. There is also an urgent need to understand more about the potential effects of recreation on invertebrates, fish, reptiles, and amphibians. We found only two articles on amphibians, but their known sensitivity to human disturbance [67] highlights the need to understand whether and how recreation affects them. Current research on recreation effects on animals does not include many species of urgent conservation concern; only about 10% of species studied are globally threatened (IUCN status of critically endangered, endangered, or vulnerable). Recreation may not be the primary reason for their endangerment, but it is a threat worth understanding because the disturbance may take place in the very protected areas designated to conserve these species. Finally, relatively few articles (31.0%) examined more than one species, and studies of species from multiple trophic levels were especially rare (3.6%). More research is needed on community-level effects of recreation, including potential cascading effects [68].

Examination of the study designs of the included articles revealed some notable trends. A fairly high percentage (30%) of articles included an experimental component; most of these were recreation treatments applied in order to compare behavioral responses. Over 80% of results examined recreation as a categorical variable, typically with three or fewer levels (e.g., low vs. high recreation activity). Though a categorical approach is simpler to implement and analyze, it limits the ability of researchers to evaluate how responses may change with different recreation intensities. It has proven difficult to develop hypothesized response curves representing how animals respond to increasing levels of recreational use due to the diversity of responses [69]. Future research should measure recreation across intensity gradients to help verify the existence of thresholds and the shape of these relationships.

Most (59%) of the effects of recreation on animals documented in the reviewed articles were negative effects. This was particularly true for reptiles, amphibians, and invertebrates, although sample sizes were low. Among invertebrate Classes, Anthozoa (corals) frequently had physical damage or reduced abundance in areas frequented by recreational divers [70,71]. Though the rate of negative effects was generally lower for birds, mammals, and fish, some lower taxonomic groups had more evidence for negative effects of recreation. For example, Order Accipitriformes (e.g., eagles, hawks) had more evidence for negative effects compared to other bird Orders, consistent with a prior meta-analysis of human disturbance on nesting birds of prey [41]. Family Charadriidae (e.g., plovers, lapwings) also had considerable evidence for negative effects of recreation, which parallels a recent study that found that species from this Order (Charadriiformes) were more frequently threatened by tourism than other bird Orders [19]. Of the mammals, Order Artiodactyla (e.g., deer, bison) had substantial evidence for negative effects, mostly consisting of behavioral responses to recreation activity. Many researchers have investigated factors that influence ungulate flight responses, including speed of approach, animal and human group size, and habitat type [43,45]. For fish, several studies found negative physiological effects of wildlife viewing on Class Chondrichthyes (e.g., sharks, stingrays; [72,73]), and negative effects of diving on fish communities [70].

Evidence for positive effects of recreational activity was much less common. Birds, particularly corvids, had more evidence for positive effects compared to most other broad taxonomic groups. Many corvids are urban adaptors [74], and several studies found that they quickly habituate to human disturbance, allowing them to tolerate or even thrive in the presence of recreationists [75,76], sometimes at the expense of other species [77]. Mammals also had a relatively high rate of positive effects. Of the mammal Orders, rodents had the most evidence for positive effects; all but one of these effects were behavioral and most resulted from habituation (e.g., reduced flight responses in areas with higher levels of recreation; [78,79]. Habituation to recreation was discussed in many (39.4%) of the included articles and typically resulted in positive responses in our coding system (e.g., reduced flight initiation distances in habituated animals), but whether habituation is a beneficial outcome for animals (e.g., by reducing costly behavioral responses to humans) is unclear and warrants further study [80,81].

We found that non-motorized activities had more evidence for negative effects than motorized activities. Motorized activities are often expected to be more harmful to animals because of vehicle speed and noise [43], but our results suggest the opposite across a wide range of study locations and taxa. A few articles directly compared motorized and non-motorized activities; four mammals (guanaco *Lama guanicoe*, wolverine *Gulo gulo*, coyote *Canis latrans*, and bobcat *Lynx rufus*) showed behavioral or occurrence responses to non-motorized but not to motorized recreation [22,82,83], whereas the reverse was found for Hector’s dolphin (*Cephalorhynchus hectori*) behavior [84] and ghost crab (*Ocypode quadrata*) abundance [85]. However, motorized activities often cover larger spatial extents than non-motorized activities, and since most studies did not compare effects across multiple spatial scales, it is possible that their impact has been underestimated. Additionally, motorized vehicles can also cause other types of harm not explored here, such as soil loss and vegetation disturbance [86]. A meta-analysis designed to explicitly compare the magnitude of effects of motorized and non-motorized recreation would be a valuable contribution to the literature.

Our results also suggest that winter terrestrial activities have greater evidence for effects on animals than summer terrestrial or aquatic activities, though the number of articles was small. A recent review of winter recreation effects on animals [39] supports this conclusion, finding that over half of the reviewed articles reported overall detrimental effects, particularly on birds and on species richness and diversity. There are several possible explanations for this result. Movement away from recreationists may be more energetically costly in snowy conditions [87]. For many species, food availability and quality is lower during winter [82,88], limiting their ability to relocate to avoid areas with human activity. There could also be habitat effects since vegetation in alpine and sub-alpine environments regenerates slowly, so habitat degradation caused by winter recreation could be more severe than that caused by other recreational activities in more temperate climates [39,89].

Overall, authors observed individual-level (behavioral and physiological) and community-level effects more frequently than most population-level (occurrence, abundance, and reproduction) effects. Though rarely measured, negative effects of recreation on survival–a particularly important response to understand for conservation purposes–were observed 1.4 times more frequently than the next highest response types (physiology and occurrence). Behavioral metrics, which were studied far more often than other types of responses, may be popular because they can be simpler to measure and have been proposed as a proxy for demographic parameters [90]. Nonetheless, behavioral metrics may not reflect the true population consequences of anthropogenic disturbance [91]. Study duration can also influence conclusions; one long-term study found that low-level recreation had an effect on dolphin habitat use that was not observed in a short-term behavioral study [81,92], while another found that short-term behavioral responses did not result in changes in the distribution or relative abundance of waterbirds [93].

Though most articles documented recreation effects, few presented specific, practical steps to minimize impacts. About 40% of the articles did not describe any management or mitigation actions, and many more contained only vague suggestions. We see a strong need for empirical tests of the effectiveness of management actions, which were rare. Encouraging examples of successful mitigation actions do exist, such as educating divers about avoiding damage to coral reefs [94], using volunteers to deter harassment of fur seals [95], and installing fences to establish disturbance-free areas [96,97]. This type of practical evaluation of management strategies is critical in assessing the ability of protected areas to meet demands for both recreational opportunities and the conservation of biodiversity. Interviewing practitioners would be a useful direction for future research in order to assess the type and extent of management strategies currently being employed. Even where management recommendations are provided in the scientific literature, it is unclear to what extent they are received by protected area managers [98]; a search of unpublished reports and other communications on the subject would help inform how well conservation scientists are reaching decision-makers.

The effects of recreation on animals is still a relatively unknown and low-profile topic in the conservation science literature, despite growing evidence that detrimental impacts can occur from a wide variety of recreational activities. Further, biophysical disturbances associated with recreation and tourism–including habitat conversion for roads and resorts, pollution from vehicles, and the spread of invasive species–are likely to have additional effects [19], increasing the overall impact of the recreation and tourism industry. Recreation effects may also act synergistically with other threats to biodiversity such as urbanization and land-use change [18], which may result in increased access for recreation. This is a troubling problem for managers and conservation practitioners, since recreation is an integral part of protected areas worldwide [12]. Finding an appropriate balance between biodiversity conservation and outdoor recreation is complicated, especially since impacts vary among species and recreation activities. We must start by simply acknowledging that these uses are not necessarily compatible for all species, in all locations. This will make it easier to justify additional research on this topic, establish restrictions on recreation, and encourage changes in the behavior of recreationists, leading to improved conservation outcomes.

## Supporting Information

S1 Appendix Articles about recreation effects on animals included in the literature review. (DOCX)

S1 File Access database containing information extracted from reviewed papers. (ACCDB)

S1 Table PRISMA checklist. (DOC)
