# Supplementary material for: Effects of Recreation on Animals Revealed as Widespread through a Global Systematic Review
Source: PLoS One. 2016 Dec 8;11(12):e0167259. doi: 10.1371/journal.pone.0167259 (PMC5145168; doi:10.1371/journal.pone.0167259)
Supplement: S1 Appendix — (DOCX) [file pone.0167259.s001.docx]

**File S1. Articles about recreation effects on animals included in the systematic review**

Addessi, L. 1994. Human disturbance and long-term changes on a rocky intertidal community. Ecological Applications **4**:786–797.

Aguilar-Melo AR, Andresen E, Cristóbal-Azkarate J, Arroyo-Rodríguez V, Chavira R, Schondube J, Serio-Silva JC, Cuarón AD. 2013. Behavioral and physiological responses to subgroup size and number of people in howler monkeys inhabiting a forest fragment used for nature-based tourism. American Journal of Primatology **75**:1108–1116.

Alomar C, Vázquez-Luis M, Magraner K, Lozano L, Deudero S. 2015. Evaluating stable isotopic signals in bivalve Pinna nobilis under different human pressures. Journal of Experimental Marine Biology and Ecology **467**:77–86.

Amo, L., P. López, and J. Martin. 2006. Nature-based tourism as a form of predation risk affects body condition and health state of *Podarcis muralis* lizards. Biological Conservation **131**:402–409.

Arlettaz, R., P. Patthey, M. Baltic, T. Leu, M. Schaub, R. Palme, and S. Jenni-Eiermann. 2007. Spreading free-riding snow sports represent a novel serious threat for wildlife. Proceedings of the Royal Society B **274**:1219–1224.

Arlettaz R, Nusslé S, Baltic M, Vogel P, Palme R, Jenni-Eiermann S, Patthey P, Genoud M. 2015. Disturbance of wildlife by outdoor winter recreation: allostatic stress response and altered activity–energy budgets. Ecological Applications **25**:1197–1212.

Ayres, E., J. N. Nkem, D. H. Wall, B. J. Adams, J. E. Barrett, E. J. Broos, A. N. Parsons, L. E. Powers, B. L. Simmons, and R. A. Virginia. 2008. Effects of human trampling on populations of soil fauna in the McMurdo dry valleys, Antarctica. Conservation Biology **22**:1544–1551.

Banks, P. B., and J. V. Bryant. 2007. Four-legged friend or foe? Dog walking displaces native birds from natural areas. Biology Letters **3**:611–613.

Barros, F. 2001. Ghost crabs as a tool for rapid assessment of human impacts on exposed sandy beaches. Biological Conservation **97**:399–404.

Baudains, T. P., and P. Lloyd. 2007. Habituation and habitat changes can moderate the impacts of human disturbance on shorebird breeding performance. Animal Conservation **10**:400–407.

Beale, C. M., and P. Monaghan. 2005. Modeling the effects of limiting the number of visitors on failure rates of seabird nests. Conservation Biology **19**:2015–2019.

Behie, A. M., M. S. M. Pavelka, and C. A. Chapman. 2010. Sources of variation in fecal cortisol levels in howler monkeys in Belize. American Journal of Primatology **72**:600–606.

Bejder, L., S. M. Dawson, and J. A. Harraway. 1999. Responses by Hector’s dolphins to boats and swimmers in Porpoise Bay, New Zealand. Marine Mammal Science **15**:738–750.

Bejder, L., A. Samuels, H. Whitehead, and N. Gales. 2006a. Interpreting short-term behavioural responses to disturbance within a longitudinal perspective. Animal Behaviour **72**:1149–1158.

Bejder, L., A. Samuels, H. Whitehead, N. Gales, J. Mann, R. Connor, M. Heithaus, J. Watson-Capps, C. Flaherty, and M. Krützen. 2006b. Decline in relative abundance of bottlenose dolphins exposed to long-term disturbance. Conservation Biology **20**:1791–1798.

Berman, C. M., J. Li, H. Ogawa, C. Ionica, and H. Yin. 2007. Primate tourism, range restriction, and infant risk among *Macaca thibetana* at Mt. Huangshan, China. International Journal of Primatology **28**:1123–1141.

Bhattacharya, T., T. Bashir, K. Poudyal, S. Sathyakumar, and G. K. Saha. 2012. Distribution, occupancy and activity patterns of goral (*Nemorhaedus goral*) and serow (*Capricornis thar*) in Khangchendzonga Biosphere Reserve, Sikkim, India. Mammal Study **37**:173–181.

Bishop A, Pomeroy P, Twiss S. 2015. Breeding male grey seals exhibit similar activity budgets across varying exposures to human activity. Marine Ecology Progress Series **527**:247–259.

Bishop, M. J. 2008. Displacement of epifauna from seagrass blades by boat wake. Journal of Experimental Marine Biology and Ecology **354**:111–118.

Blom, A., C. Cipolletta, A. M. H. Brunsting, and H. H. T. Prins. 2004. Behavioral responses of gorillas to habituation in the Dzanga-Ndoki National Park, Central African Republic. International Journal of Primatology **25**:179–196.

Bolduc, F., and M. Guillemette. 2003. Human disturbance and nesting success of common eiders: interaction between visitors and gulls. Biological Conservation **110**:77–83.

Borkowski, J. 2001. Flight behaviour and observability in human-disturbed sika deer. Acta Theriologica **46**:195–206.

Borkowski, J. J., P. J. White, R. A. Garrott, T. Davis, A. R. Hardy, and D. J. Reinhart. 2006. Behavioral responses of bison and elk in Yellowstone to snowmobiles and snow coaches. Ecological Applications **16**:1911–1925.

Bowen, K. D., and F. J. Janzen. 2008. Human recreation and the nesting ecology of a freshwater turtle (*Chrysemys picta*). Chelonian Conservation and Biology **7**:95–100.

Braunisch, V., P. Patthey, and R. Arlettaz. 2011. Spatially explicit modeling of conflict zones between wildlife and snow sports: prioritizing areas for winter refuges. Ecological Applications **21**:955–967.

Bright, A., G. R. Reynolds, J. Innes, and J. R. Waas. 2003. Effects of motorised boat passes on the time budgets of New Zealand dabchick, *Poliocephalus rufopectus*. Wildlife Research **30**:237–244.

Bright, A., J. R. Waas, and J. Innes. 2004. Correlations between human-made structures, boat-pass frequency and the number of New Zealand dabchicks (*Poliocephalus rufopectus*) on the Rotorua Lakes, New Zealand. New Zealand Journal of Ecology **28**:137–142.

Burger, J., and M. Gochfeld. 2007. Responses of emperor penguins (*Aptenodytes forsteri*) to encounters with ecotourists while commuting to and from their breeding colony. Polar Biology **30**:1303–1313.

Burger, J., M. Gochfeld, C. D. Jenkins, and F. Lesser. 2010. Effect of approaching boats on nesting black skimmers: using response distances to establish protective buffer zones. Journal of Wildlife Management **74**:102–108.

Cardoni, D. A., M. Favero, and J. P. Isacch. 2008. Recreational activities affecting the habitat use by birds in Pampa’s wetlands, Argentina: implications for waterbird conservation. Biological Conservation **141**:797–806.

Cassini, M. H., D. Szteren, and E. Fernández-Juricic. 2004. Fence effects on the behavioural responses of South American fur seals to tourist approaches. Journal of Ethology **22**:127–133.

Christiansen F, Rasmussen M, Lusseau D. 2013. Whale watching disrupts feeding activities of minke whales on a feeding ground. Marine Ecology Progress Series **478**:239–251.

Christiansen F, Rasmussen MH, Lusseau D. 2013. Inferring activity budgets in wild animals to estimate the consequences of disturbances. Behavioral Ecology **24**:1415–1425.

Christiansen F, Rasmussen MH, Lusseau D. 2014. Inferring energy expenditure from respiration rates in minke whales to measure the effects of whale watching boat interactions. Journal of Experimental Marine Biology and Ecology **459**:96–104.

Claudet, J., P. Lenfant, and M. Schrimm. 2010. Snorkelers impact on fish communities and algae in a temperate marine protected area. Biodiversity and Conservation **19**:1649–1658.

Cobley, N. D., and J. R. Shears. 1999. Breeding performance of gentoo penguins (*Pygoscelis papua*) at a colony exposed to high levels of human disturbance. Polar Biology **21**:355–360.

Coleman TH, Schwartz CC, Gunther KA, Creel S. 2013. Grizzly bear and human interaction in Yellowstone National Park: An evaluation of bear management areas. The Journal of Wildlife Management **77**:1311–1320.

Coleman TH, Schwartz CC, Gunther KA, Creel S. 2013. Influence of overnight recreation on grizzly bear movement and behavior in Yellowstone National Park. Ursus **24**:101–110.

Colman, J. E., B. W. Jacobsen, and E. Reimers. 2001. Summer response distances of Svalbard reindeer *Rangifer tarandus platyrhynchus* to provocation by humans on foot. Wildlife Biology **7**:275–284.

Coma, R., E. Pola, M. Ribes, and M. Zabala. 2004. Long-term assessment of temperate octocoral mortality patterns, protected vs. unprotected areas. Ecological Applications **14**:1466–1478.

Comor, V., J. Orgeas, P. Ponel, C. Rolando, and Y. R. Delettre. 2008. Impact of anthropogenic disturbances on beetle communities of French Mediterranean coastal dunes. Biodiversity and Conservation **17**:1837–1852.

Constantine, R. 2001. Increased avoidance of swimmers by wild bottlenose dolphins (*Tursiops truncatus*) due to long-term exposure to swim-with-dolphin tourism. Marine Mammal Science **17**:689–702.

Constantine, R., D. H. Brunton, and T. Dennis. 2004. Dolphin-watching tour boats change bottlenose dolphin (*Tursiops truncatus*) behaviour. Biological Conservation **117**:299–307.

Cooper, C. A., A. J. Neff, D. P. Poon, and G. R. Smith. 2008. Behavioral responses of Eastern gray squirrels in suburban habitats differing in human activity levels. Northeastern Naturalist **15**:619–625.

Cornelisse TM, Duane TP. 2013. Effects of Knowledge of an Endangered Species on Recreationists’ Attitudes and Stated Behaviors and the Significance of Management Compliance for Ohlone Tiger Beetle Conservation. Conservation Biology **27**:1449–1457.

Cornelius, C., S. A. Navarrete, and P. A. Marquet. 2001. Effects of human activity on the structure of coastal marine bird assemblages in central Chile. Conservation Biology **15**:1396–1404.

Costello CM, Cain SI, Nielson RM, Servheen C, Schwartz CC. 2013. Response of American black bears to the non-motorized expansion of a road corridor in Grand Teton National Park. Ursus **24**:54–69.

Cowling M, Kirkwood R, Boren L, Sutherland D, Scarpaci C. 2015. The effects of vessel approaches on the New Zealand fur seal (Arctocephalus forsteri) in the Bay of Plenty, New Zealand. Marine Mammal Science **31**:501–519.

Cunha, A. A. 2010. Negative effects of tourism in a Brazilian Atlantic forest National Park. Journal for Nature Conservation **18**:291–295.

Dans, S. L., M. Degrati, S. N. Pedraza, and E. A. Crespo. 2012. Effects of tour boats on dolphin activity examined with sensitivity analysis of Markov chains. Conservation Biology **26**:708–716.

De la Torre, S., C. T. Snowdon, and M. Bejarano. 2000. Effects of human activities on wild pygmy marmosets in Ecuadorian Amazonia. Biological Conservation **94**:153–163.

Deluca WV, King DI. 2014. Influence of hiking trails on montane birds. The Journal of Wildlife Management **78**:494–502.

Devney, C. A., and B. C. Congdon. 2009. Testing the efficacy of a boundary fence at an important seabird breeding colony and key tourist destination. Wildlife Research **36**:353–360.

Diego-Rasilla, F. J. 2003. Human influence on the tameness of wall lizard, *Podarcis muralis*. Italian Journal of Zoology **70**:225–228.

Duchesne, M., S. D. Côté, and C. Barrette. 2000. Responses of woodland caribou to winter ecotourism in the Charlevoix Biosphere Reserve, Canada. Biological Conservation **96**:311–317.

Dyck, M. G., and R. K. Baydack. 2004. Vigilance behaviour of polar bears (*Ursus maritimus*) in the context of wildlife-viewing activities at Churchill, Manitoba, Canada. Biological Conservation **116**:343–350.

El Alami A, Chait A. 2013. Comparison of the terrestriality of Barbary macaques (Macaca sylvanus) between tourist and wild sites in the region of Ouzoud, Morocco. Mammalia **78**:539–542.

El Alami, A., E. Van Lavieren, A. Rachida, and A. Chait. 2012. Differences in activity budgets and diet Between semiprovisioned and wild-feeding groups of the endangered Barbary macaque (*Macaca sylvanus*) in the Central High Atlas Mountains, Morocco. American Journal of Primatology **74**:210–216.

Ellenberg, U., T. Mattern, and P. J. Seddon. 2009. Habituation potential of yellow-eyed penguins depends on sex, character and previous experience with humans. Animal Behaviour **77**:289–296.

Elmeligi S, Shultis J. 2015. Impacts of Boat-Based Wildlife Viewing in the K’tzim-a-Deen Inlet on Grizzly Bear (Ursus arctos) Behavior. Natural Areas Journal **35**:404–415.

Fairbanks, W. S., and R. Tullous. 2002. Distribution of pronghorn (*Antilocapra americana* Ord) on Antelope Island State Park, Utah, USA, before and after establishment of recreational trails. Natural Areas Journal **22**:277–282.

Fernández-Juricic, E., M. D. Jimenez, and E. Lucas. 2002. Factors affecting intra- and inter-specific variations in the difference between alert distances and flight distances for birds in forested habitats. Canadian Journal of Zoology/Revue Canadienne de Zoologie **80**:1212–1220.

Fernández-Juricic, E., R. Vaca, and N. Schroeder. 2004. Spatial and temporal responses of forest birds to human approaches in a protected area and implications for two management strategies. Biological Conservation **117**:407–416.

Fernández-Juricic, E., M. P. Venier, D. Renison, and D. T. Blumstein. 2005. Sensitivity of wildlife to spatial patterns of recreationist behavior: a critical assessment of minimum approaching distances and buffer areas for grassland birds. Biological Conservation **125**:225–235.

Fernández-Juricic, E., E. F. Zahn, T. Parker, T. Stankowich, and others. 2009. California’s endangered Belding’s savannah sparrow (*Passerculus sandwichensis beldingi*): tolerance of pedestrian disturbance. Avian Conservation and Ecology-Écologie et conservation des oiseaux **4**:1.

Ficetola, G. F., R. Sacchi, S. Scali, A. Gentilli, F. De Bernardi, and P. Galeotti. 2007. Vertebrates respond differently to human disturbance: implications for the use of a focal species approach. Acta Oecologica **31**:109–118.

Finney, S. K., J. W. Pearce-Higgins, and D. W. Yalden. 2005. The effect of recreational disturbance on an upland breeding bird, the golden plover *Pluvialis apricaria*. Biological Conservation **121**:53–63.

Foroughirad V, Mann J. 2013. Long-term impacts of fish provisioning on the behavior and survival of wild bottlenose dolphins. Biological Conservation **160**:242–249.

Fowler, G. S. 1999. Behavioral and hormonal responses of Magellanic penguins (*Spheniscus magellanicus*) to tourism and nest site visitation. Biological Conservation **90**:143–149.

Galicia, E., and G. A. Baldassarre. 1997. Effects of motorized tourboats on the behavior of nonbreeding American flamingos in Yucatan, Mexico. Conservation Biology **11**:1159–1165.

Garber, S. D., and J. Burger. 1995. A 20-year study documenting the relationship between turtle decline and human recreation. Ecological Applications **5**:1151–1162.

Garrido M, Pérez-Mellado V. 2015. Human pressure, parasitism and body condition in an insular population of a Mediterranean lizard. European Journal of Wildlife Research **61**:617–621.

George, S. L., and K. R. Crooks. 2006. Recreation and large mammal activity in an urban nature reserve. Biological Conservation **133**:107–117.

Giese, M. 1996. Effects of human activity on Adelie penguin *Pygoscelis adeliae* breeding success. Biological Conservation **75**:157–164.

Gill, J. A., K. Norris, and W. J. Sutherland. 2001. The effects of disturbance on habitat use by black-tailed godwits *Limosa limosa*. Journal of Applied Ecology **38**:846–856.

Glover, H. K., M. A. Weston, G. S. Maguire, K. K. Miller, and B. A. Christie. 2011. Towards ecologically meaningful and socially acceptable buffers: response distances of shorebirds in Victoria, Australia, to human disturbance. Landscape and Urban Planning **103**:326–334.

Goldstein, M. I., A. J. Poe, L. H. Suring, R. M. Nielson, and T. L. McDonald. 2009. Brown bear den habitat and winter recreation in south-central Alaska. Journal of Wildlife Management **74**:35–42.

Griffin, S. C., T. Valois, M. L. Taper, and L. Scott Mills. 2007. Effects of tourists on behavior and demography of Olympic marmots. Conservation Biology **21**:1070–1081.

Grubb, T. G., D. K. Delaney, W. W. Bowerman, and M. R. Wierda. 2010. Golden eagle indifference to heli-Skiing and military helicopters in northern Utah. Journal of Wildlife Management **74**:1275–1285.

Guillemain, M., R. Blanc, C. Lucas, and M. Lepley. 2007. Ecotourism disturbance to wildfowl in protected areas: historical, empirical and experimental approaches in the Camargue, Southern France. Biodiversity and Conservation **16**:3633–3651.

Gutzwiller, K. J., and S. K. Riffell. 2008. Does repeated human intrusion alter use of wildland sites by red squirrels? Multiyear experimental evidence. Journal of Mammalogy **89**:374–380.

Gutzwiller, K. J., S. K. Riffell, and S. H. Anderson. 2002. Repeated human intrusion and the potential for nest predation by gray jays. The Journal of Wildlife Management **66**:372–380.

Hansen BB, Aanes R. 2014. Habituation to humans in a predator-free wild ungulate. Polar Biology **38**:145–151.

Harris G, Nielson RM, Rinaldi T, Lohuis T. 2014. Effects of winter recreation on northern ungulates with focus on moose (Alces alces) and snowmobiles. European Journal of Wildlife Research **60**:45–58.

Haskell PJ et al. 2015. Monitoring the effects of tourism on whale shark Rhincodon typus behaviour in Mozambique. Oryx **49**:492–499.

Hawkins, J. P., and C. M. Roberts. 1992. Effects of recreational SCUBA diving on fore-reef slope communities of coral reefs. Biological Conservation **62**:171–178.

Hawkins, J. P., and C. M. Roberts. 1993. Effects of recreational scuba diving on coral reefs: trampling on reef-flat communities. Journal of Applied Ecology **30**:25–30.

Hawkins, J. P., C. M. Roberts, T. V. Hof, K. D. Meyer, J. Tratalos, and C. Aldam. 1999. Effects of recreational scuba diving on Caribbean coral and fish Communities. Conservation Biology **13**:888–897.

Hayward, M., and G. Hayward. 2009. The impact of tourists on lion *Panthera leo* behaviour, stress and energetics. Acta Theriologica **54**:219–224.

Heil, L., E. Fernandez-Juricic, D. Renison, A. M. Cingolani, and D. T. Blumstein. 2007. Avian responses to tourism in the biogeographically isolated high Cordoba Mountains, Argentina. Biodiversity and Conservation **16**:1009–1026.

Heyman, W., L. Carr, and P. Lobel. 2010. Diver ecotourism and disturbance to reef fish spawning aggregations: It is better to be disturbed than to be dead. Marine Ecology Progress Series **419**:201–210.

Hillman MD, Karpanty SM, Fraser JD, Derose-Wilson A. 2015. Effects of aircraft and recreation on colonial waterbird nesting behavior. The Journal of Wildlife Management **79**:1192–1198.

Hines, K. N. 2011. Effects of ecotourism on endangered northern Bahamian rock iguanas (*Cyclura cychlura*). Herpetological Conservation and Biology **6**:250–259.

Hodgson, A. J., H. Marsh, and P. J. Corkeron. 2004. Provisioning by tourists affects the behaviour but not the body condition of Mareeba rock-wallabies (*Petrogale mareeba*). Wildlife Research **31**:451–456.

Holcomb, K., J. K. Young, and L. R. Gerber. 2009. The influence of human disturbance on California sea lions during the breeding season. Animal Conservation **12**:592–598.

Holmes, N. D. 2007. Comparing king, gentoo, and royal penguin responses to pedestrian visitation. Journal of Wildlife Management **71**:2575–2582.

Holmes, N., M. Giese, and L. K. Kriwoken. 2005. Testing the minimum approach distance guidelines for incubating Royal penguins *Eudyptes schlegeli*. Biological Conservation **126**:339–350.

Hsieh, Y.-L., Y.-S. Lin, and I.-M. Tso. 2003. Ground spider diversity in the Kenting uplifted coral reef forest, Taiwan: a comparison between habitats receiving various disturbances. Biodiversity and Conservation **12**:2173–2194.

Hsu, C., C. Chen, and H. Hsieh. 2009a. Effects of sediment compaction on macroinfauna in a protected coastal wetland in Taiwan. Marine Ecology Progress Series **375**:73–83.

Hsu, M. J., C.-C. Kao, and G. Agoramoorthy. 2009b. Interactions between visitors and Formosan macaques (*Macaca cyclopis*) at Shou-Shan Nature Park, Taiwan. American Journal of Primatology **71**:214–222.

Hulbert, I. A. R. 1990. The response of ruddy shelduck *Tadorna ferruginea* to tourist activity in the Royal Chitwan National Park of Nepal. Biological Conservation **52**:113–123.

Ikuta, L. A., and D. T. Blumstein. 2003. Do fences protect birds from human disturbance? Biological Conservation **112**:447–452.

Ilarri, M. D. I., A. T. de Souza, P. R. de Medeiros, R. G. Grempel, and I. M. de L. Rosa. 2008. Effects of tourist visitation and supplementary feeding on fish assemblage composition on a tropical reef in the Southwestern Atlantic. Neotropical Ichthyology **6**:651–656.

Iverson, J. B., S. J. Converse, G. R. Smith, and J. M. Valiulis. 2006. Long-term trends in the demography of the Allen Cays Rock Iguana (*Cyclura cychlura inornata*): human disturbance and density-dependent effects. Biological Conservation **132**:300–310.

Jayakody, S., A. M. Sibbald, I. J. Gordon, and X. Lambin. 2008. Red deer *Cervus elephus* vigilance behaviour differs with habitat and type of human disturbance. Wildlife Biology **14**:81–91.

Jayakody, S., A. M. Sibbald, R. W. Mayes, R. J. Hooper, I. J. Gordon, and X. Lambin. 2011. Effects of human disturbance on the diet composition of wild red deer (*Cervus elaphus*). European Journal of Wildlife Research **57**:939–948.

Jiménez G, Lemus JA, Meléndez L, Blanco G, Laiolo P. 2011. Dampened behavioral and physiological responses mediate birds’ association with humans. Biological Conservation **144**:1702–1711.

Kaiser, M. S., and E. K. Fritzell. 1984. Effects of river recreationists on green-backed heron behavior. The Journal of Wildlife Management **48**:561–567.

Kangas, K., M. Luoto, A. Ihantola, E. Tomppo, and P. Siikamäki. 2010. Recreation-induced changes in boreal bird communities in protected areas. Ecological Applications **20**:1775–1786.

Karp, D. S., and R. Guevara. 2011. Conversational noise reduction as a win–win for ecotourists and rain forest birds in Peru. Biotropica **43**:122–130.

Karp, D. S., and T. L. Root. 2009. Sound the stressor: how hoatzins (*Opisthocomus hoazin*) react to ecotourist conversation. Biodiversity and Conservation **18**:3733–3742.

Keeley, W. H., and M. J. Bechard. 2011. Flushing distances of ferruginous hawks nesting in rural and exurban New Mexico. Journal of Wildlife Management **75**:1034–1039.

Keller, V. E. 1991. Effects of human disturbance on eider ducklings *Somateria mollissima* in an estuarine habitat in Scotland. Biological Conservation **58**:213–228.

Kerbiriou, C., I. Le Viol, A. Robert, E. Porcher, F. Gourmelon, and R. Julliard. 2009. Tourism in protected areas can threaten wild populations: from individual response to population viability of the chough *Pyrrhocorax pyrrhocorax*. Journal of Applied Ecology **46**:657–665.

King, J. M., and J. T. Heinen. 2004. An assessment of the behaviors of overwintering manatees as influenced by interactions with tourists at two sites in central Florida. Biological Conservation **117**:227–234.

Klailova, M., C. Hodgkinson, and P. C. Lee. 2010. Behavioral responses of one western lowland gorilla (*Gorilla gorilla gorilla*) group at Bai Hokou, Central African Republic, to tourists, researchers and trackers. American Journal of Primatology **72**:897–906.

Klein, M. L., S. R. Humphrey, and H. F. Percival. 1995. Effects of ecotourism on distribution of waterbirds in a wildlife refuge. Conservation Biology **9**:1454–1465.

Krebs, J., E. C. Lofroth, and I. Parfitt. 2007. Multiscale habitat use by wolverines in British Columbia, Canada. Journal of Wildlife Management **71**:2180–2192.

Lafferty, K. D. 2001a. Disturbance to wintering western snowy plovers. Biological Conservation **101**:315–325.

Lafferty, K. D. 2001. Birds at a southern California beach: seasonality, habitat use and disturbance by human activity. Biodiversity and Conservation **10**:1949–1962.

Lafferty, K. D., D. Goodman, and C. P. Sandoval. 2006. Restoration of breeding by snowy plovers following protection from disturbance. Biodiversity and Conservation **15**:2217–2230.

Lamb, J. B., and B. L. Willis. 2011. Using coral disease prevalence to assess the effects of concentrating tourism activities on offshore reefs in a tropical marine park. Conservation Biology **25**:1044–1052.

Lamb JB, True JD, Piromvaragorn S, Willis BL. 2014. Scuba diving damage and intensity of tourist activities increases coral disease prevalence. Biological Conservation **178**:88–96.

Laroche, R. K., A. A. Kock, L. M. Dill, and W. H. Oosthuizen. 2007. Effects of provisioning ecotourism activity on the behaviour of white sharks Carcharodon carcharias. Marine Ecology Progress Series **338**:199–209.

Leighton, P. A., J. A. Horrocks, and D. L. Kramer. 2010. Conservation and the scarecrow effect: Can human activity benefit threatened species by displacing predators? Biological Conservation **143**:2156–2163.

Lemon, M., T. P. Lynch, D. H. Cato, and R. G. Harcourt. 2006. Response of travelling bottlenose dolphins (*Tursiops aduncus*) to experimental approaches by a powerboat in Jervis Bay, New South Wales, Australia. Biological Conservation **127**:363–372.

Lenth, B. E., R. L. Knight, and M. E. Brennan. 2008. The effects of dogs on wildlife communities. Natural Areas Journal **28**:218–227.

Lima AC, Assis J, Sayanda D, Sabino J, Oliveira RF, Lima AC, Assis J, Sayanda D, Sabino J, Oliveira RF. 2014. Impact of ecotourism on the fish fauna of Bonito region (Mato Grosso do Sul State, Brazil): ecological, behavioural and physiological measures. Neotropical Ichthyology **12**:133–143.

Lott, D. F., and M. McCoy. 1995. Asian rhinos *Rhinoceros unicornis* on the run? Impact of tourist visits on one population. Biological Conservation **73**:23–26.

Lowe A, Rogers AC, Durrant KL. 2014. Effect of human disturbance on long-term habitat use and breeding success of the European Nightjar, Caprimulgus europaeus. Avian Conservation and Ecology **9**. Available from http://www.ace-eco.org/vol9/iss2/art6/ (accessed March 28, 2016).

Lundquist D, Sironi M, Würsig B, Rowntree V, Martino J, Lundquist L. 2013. Response of southern right whales to simulated swim-with-whale tourism at Península Valdés, Argentina. Marine Mammal Science **29**:E24–E45.

Luo J, Jiang T, Lu G, Wang L, Wang J, Feng J. 2013. Bat conservation in China: should protection of subterranean habitats be a priority? Oryx **47**:526–531.

Lusseau, D. 2003a. Male and female bottlenose dolphins *Tursiops spp*. have different strategies to avoid interactions with tour boats in Doubtful Sound, New Zealand. Marine Ecology Progress Series **257**:267–274.

Lusseau, D. 2003b. Effects of tour boats on the behavior of bottlenose dolphins: using Markov chains to model anthropogenic impacts. Conservation Biology **17**:1785–1793.

Lusseau, D. 2004. The hidden cost of tourism: detecting long-term effects of tourism using behavioral information. Ecology and Society **9**:2.

Lusseau, D. 2005. Residency pattern of bottlenose dolphins *Tursiops spp*. in Milford Sound, New Zealand, is related to boat traffic. Marine Ecology Progress Series **295**:265–272.

Lusseau, D. 2006. The short-term behavioral reactions of bottlenose dolphins to interactions with boats in Doubtful Sound, New Zealand. Marine Mammal Science **22**:802–818.

Lynch, H. J., W. F. Fagan, and R. Naveen. 2010. Population trends and reproductive success at a frequently visited penguin colony on the western Antarctic peninsula. Polar Biology **33**:493–503.

Lyons PJ et al. 2015. The effect of recreational SCUBA divers on the structural complexity and benthic assemblage of a Caribbean coral reef. Biodiversity and Conservation **24**:3491–3504.

Madsen, J. 1998. Experimental refuges for migratory waterfowl in Danish wetlands. I. Baseline assessment of the disturbance effects of recreational activities. Journal of Applied Ecology **35**:386–397.

Mainini, B., P. Neuhaus, and P. Ingold. 1993. Behaviour of marmots *Marmota marmota* under the influence of different hiking activities. Biological Conservation **64**:161–164.

Maljković, A., and I. M. Côté. 2011. Effects of tourism-related provisioning on the trophic signatures and movement patterns of an apex predator, the Caribbean reef shark. Biological Conservation **144**:859–865.

Mallord, J. W., P. M. Dolman, A. F. Brown, and W. J. Sutherland. 2007. Linking recreational disturbance to population size in a ground-nesting passerine. Journal of Applied Ecology **44**:185–195.

Malo, J., P. Acebes, and J. Traba. 2011. Measuring ungulate tolerance to human with flight distance: a reliable visitor management tool? Biodiversity and Conservation **20**:3477–3488.

Mann, S. L., R. J. Steidl, and V. M. Dalton. 2002. Effects of cave tours on breeding *Myotis velifer*. The Journal of Wildlife Management **66**:618–624.

Marchand P, Garel M, Bourgoin G, Dubray D, Maillard D, Loison A. 2014. Impacts of tourism and hunting on a large herbivore’s spatio-temporal behavior in and around a French protected area. Biological Conservation **177**:1–11.

Maréchal, L., S. Semple, B. Majolo, M. Qarro, M. Heistermann, and A. MacLarnon. 2011. Impacts of tourism on anxiety and physiological stress levels in wild male Barbary macaques. Biological Conservation **144**:2188–2193.

Markovchick-Nicholls, L., H. M. Regan, D. H. Deutschman, A. Widyanata, B. Martin, L. Noreke, and T. Ann Hunt. 2008. Relationships between human disturbance and wildlife land use in urban habitat fragments. Conservation Biology **22**:99–109.

Martin, J., L. de Neve, J. Antonio, V. Polo, and M. Soler. 2004. Factors affecting the escape behaviour of juvenile chinstrap penguins, *Pygoscelis antarctica*, in response to human disturbance. Polar Biology **27**:775–781.

Martin, J., P. L. Fackler, J. D. Nichols, M. C. Runge, C. L. McIntyre, B. L. Lubow, M. C. McCluskie, and J. A. Schmutz. 2011. An adaptive-management framework for optimal control of hiking near golden eagle nests in Denali National Park. Conservation Biology **25**:316–323.

Martinez, E., M. B. Orams, M. D. M. Pawley, and K. A. Stockin. 2012. The use of auditory stimulants during swim encounters with Hector’s dolphins (*Cephalorhynchus hectori hectori*) in Akaroa Harbour, New Zealand. Marine Mammal Science **28**:E295–E315.

Massé S, Dussault C, Dussault C, Ibarzabal J. 2014. How artificial feeding for tourism-watching modifies black bear space use and habitat selection. The Journal of Wildlife Management **78**:1228–1238.

McCarthy KP, Fletcher RJ. 2015. Does hunting activity for game species have indirect effects on resource selection by the endangered Florida panther? Animal Conservation **18**:138–145.

McClung, M. R., P. J. Seddon, M. Massaro, and A. N. Setiawan. 2004. Nature-based tourism impacts on yellow-eyed penguins *Megadyptes antipodes*: does unregulated visitor access affect fledging weight and juvenile survival? Biological Conservation **119**:279–285.

McGrann, M. C., G. R. Wright, R. J. Dial, and A. M. McGrann. 2006. Off-highway vehicle impact on the flat-tailed horned lizard, *Phrynosoma mcallii*, in the Colorado Desert of Southern California. California Fish and Game **92**:67–80.

McKinney T. 2014. Species-Specific Responses to Tourist Interactions by White-Faced Capuchins (Cebus imitator) and Mantled Howlers (Alouatta palliata) in a Costa Rican Wildlife Refuge. International Journal of Primatology **35**:573–589.

McMillan, M. A., J. C. Nekola, and D. W. Larson. 2003. Effects of rock climbing on the land snail community of the Niagara escarpment in southern Ontario, Canada. Conservation Biology **17**:616–621.

Ménard N, Foulquier A, Vallet D, Qarro M, Le Gouar P, Pierre J-S. 2014. How tourism and pastoralism influence population demographic changes in a threatened large mammal species. Animal Conservation **17**:115–124.

Milazzo, M. 2011. Evaluation of a behavioural response of Mediterranean coastal fishes to novel recreational feeding situation. Environmental Biology of Fishes **91**:127–132.

Milazzo, M., I. Anastasi, and T. J. Willis. 2006. Recreational fish feeding affects coastal fish behavior and increases frequency of predation on damselfish *Chromis chromis* nests. Marine Ecology Progress Series **310**:165–172.

Miller, J. R., and N. T. Hobbs. 2000. Recreational trails, human activity, and nest predation in lowland riparian areas. Landscape and Urban Planning **50**:227–236.

Miller, S. G., R. L. Knight, and C. K. Miller. 1998. Influence of recreational trails on breeding bird communities. Ecological Applications **8**:162–169.

Moore, M. J. C., and R. A. Seigel. 2006. No place to nest or bask: effects of human disturbance on the nesting and basking habits of yellow-blotched map turtles (*Graptemys flavimaculata*). Biological Conservation **130**:386–393.

Morrison, M. L., R. J. Young, J. S. Romsos, and R. Golightly. 2011. Restoring forest raptors: influence of human disturbance and forest condition on Northern Goshawks. Restoration Ecology **19**:273–279.

Moss R, Leckie F, Biggins A, Poole T, Baines D, Kortland K. 2014. Impacts of Human Disturbance on Capercaillie Tetrao urogallus Distribution and Demography in Scottish Woodland. Wildlife Biology **20**:1–18.

Müllner, A., K. Eduard Linsenmair, and M. Wikelski. 2004. Exposure to ecotourism reduces survival and affects stress response in hoatzin chicks (*Opisthocomus hoazin*). Biological Conservation **118**:549–558.

Murph JH, Faulkes Z. 2013. Abundance and Size of Sand Crabs, Lepidopa benedicti (Decapoda: Albuneidae), In Southern Texas. The Southwestern Naturalist **58**:431–434.

Naylor, L. M., M. J. Wisdom, and R. G. Anthony. 2009. Behavioral responses of North American elk to recreational activity. Journal of Wildlife Management **73**:328–338.

Neuhaus, P., and B. Mainini. 1998. Reactions and adjustment of adult and young alpine marmots *Marmota marmota* to intense hiking activities. Wildlife Biology **4**:119–123.

Neumann, W., G. Ericsson, and H. Dettki. 2010. Does off-trail backcountry skiing disturb moose? European Journal of Wildlife Research **56**:513–518.

Nyhof PE, Trulio L. 2015. Basking Western Pond Turtle Response to Recreational Trail Use in Urban California. Chelonian Conservation and Biology **14**:182–184.

Ordiz A, Støen O-G, Sæbø S, Sahlén V, Pedersen BE, Kindberg J, Swenson JE. 2013. Lasting behavioural responses of brown bears to experimental encounters with humans. Journal of Applied Ecology **50**:306–314.

Osinga, N., S. B. Nussbaum, P. M. Brakefield, and H. A. Udo de Haes. 2012. Response of common seals (*Phoca vitulina*) to human disturbances in the Dollard estuary of the Wadden Sea. Mammalian Biology - Zeitschrift für Säugetierkunde **77**:281–287.

Paksuz, S., and B. Özkan. 2012. The protection of the bat community in the Dupnisa Cave System, Turkey, following opening for tourism. Oryx **46**:130–136.

Papouchis, C. M., F. J. Singer, and W. B. Sloan. 2001. Responses of desert bighorn sheep to increased human recreation. Journal of Wildlife Management **65**:573–582.

Parent, C., and P. J. Weatherhead. 2000. Behavioral and life history responses of eastern Massasauga rattlesnakes (*Sistrurus catenatus catenatus*) to human disturbance. Oecologia **125**:170–178.

Patterson, M. E., J. D. Fraser, and J. W. Roggenbuck. 1991. Factors affecting piping plover productivity on Assateague Island. Journal of Wildlife Management **55**:525–531.

Patthey, P., S. Wirthner, N. Signorell, and R. Arlettaz. 2008. Impact of outdoor winter sports on the abundance of a key indicator species of alpine ecosystems. Journal of Applied Ecology **45**:1704–1711.

Pavez G, Muñoz L, Barilari F, Sepúlveda M. 2015. Variation in behavioral responses of the South American sea lion to tourism disturbance: Implications for tourism management. Marine Mammal Science **31**:427–439.

Pęksa Ł, Ciach M. 2015. Negative effects of mass tourism on high mountain fauna: the case of the Tatra chamois Rupicapra rupicapra tatrica. Oryx **49**:500–505.

Peters, K. A., and D. L. Otis. 2007. Shorebird roost-site selection at two temporal scales: is human disturbance a factor? Journal of Applied Ecology **44**:196–209.

Peters KJ, Parra GJ, Skuza PP, Möller LM. 2013. First insights into the effects of swim-with-dolphin tourism on the behavior, response, and group structure of southern Australian bottlenose dolphins. Marine Mammal Science **29**:E484–E497.

Pfeiffer, S., and H.-U. Peter. 2004. Ecological studies toward the management of an Antarctic tourist landing site (Penguin Island, South Shetland Islands). Polar Record **40**:345–353.

Phillips, G. E., and A. W. Alldredge. 2000. Reproductive success of elk following disturbance by humans during calving season. Journal of Wildlife Management **64**:521–530.

Picciulin, M., L. Sebastianutto, A. Codarin, A. Farina, and E. A. Ferrero. 2010. In situ behavioural responses to boat noise exposure of *Gobius cruentatus* (Gmelin, 1789; fam. Gobiidae) and *Chromis chromis* (Linnaeus, 1758; fam. Pomacentridae) living in a marine protected area. Journal of Experimental Marine Biology and Ecology **386**:125–132.

Pineiro, A., I. Barja, G. Silvan, and J. Carlos Illera. 2012. Effects of tourist pressure and reproduction on physiological stress response in wildcats: management implications for species conservation. Wildlife Research **39**:532–539.

Preisler, H. K., A. A. Ager, and M. J. Wisdom. 2006. Statistical methods for analysing responses of wildlife to human disturbance. Journal of Applied Ecology **43**:164–172.

Randler, C. 2006. Disturbances by dog barking increase vigilance in coots *Fulica atra*. European Journal of Wildlife Research **52**:265–270.

Reed, S. E., and A. M. Merenlender. 2008. Quiet, nonconsumptive recreation reduces protected area effectiveness. Conservation Letters **1**:146–154.

Reed, S. E., and A. M. Merenlender. 2011. Effects of management of domestic dogs and recreation on carnivores in protected areas in Northern California. Conservation Biology **25**:504–513.

Rehnus M, Wehrle M, Palme R. 2014. Mountain hares Lepus timidus and tourism: stress events and reactions. Journal of Applied Ecology **51**:6–12.

Reimers, E., S. Eftestøl, and J. E. Colman. 2003. Behavior responses of wild reindeer to direct provocation by a snowmobile or skier. Journal of Wildlife Management **67**:747–754.

Reimers, E., F. L. Miller, S. Eftestøl, J. E. Colman, and B. Dahle. 2006. Flight by feral reindeer Rangifer *tarandus tarandus* in response to a directly approaching human on foot or on skis. Wildlife Biology **12**:403–413.

Riffell, S. K., K. J. Gutzwiller, and S. H. Anderson. 1996. Does repeated human intrusion cause cumulative declines in avian richness and abundance? Ecological Applications **6**:492–505.

Rode, K. D., S. D. Farley, J. Fortin, and C. T. Robbins. 2007. Nutritional consequences of experimentally introduced tourism in brown bears. Journal of Wildlife Management **71**:929–939.

Rode, K. D., S. D. Farley, and C. T. Robbins. 2006. Behavioral responses of brown bears mediate nutritional effects of experimentally introduced tourism. Biological Conservation **133**:70–80.

Rode, K. D., S. D. Farley, and C. T. Robbins. 2006. Sexual dimorphism, reproductive strategy, and human activities determine resource use by brown bears. Ecology **87**:2636–2646.

Rodgers, J. A., and S. T. Schwikert. 2002. Buffer-zone distances to protect foraging and loafing waterbirds from disturbance by personal watercraft and outboard-powered boats. Conservation Biology **16**:216–224.

Rodríguez-Prieto, I., and E. Fernández-Juricic. 2005. Effects of direct human disturbance on the endemic Iberian frog *Rana iberica* at individual and population levels. Biological Conservation **123**:1–9.

Rodriguez-Prieto, I., E. Fernández-Juricic, J. Martín, and Y. Regis. 2009. Antipredator behavior in blackbirds: habituation complements risk allocation. Behavioral Ecology **20**:371–377.

Romero, L. M., and M. Wikelski. 2002. Exposure to tourism reduces stress-induced corticosterone levels in Galápagos marine iguanas. Biological Conservation **108**:371–374.

Rösner S, Mussard-forster E, Lorenc T, Müller J. 2014. Recreation shapes a “landscape of fear” for a threatened forest bird species in Central Europe. Landscape Ecology **29**:55–66.

Rouphael, A. B., and G. J. Inglis. 2001. “Take only photographs and leave only footprints”?: An experimental study of the impacts of underwater photographers on coral reef dive sites. Biological Conservation **100**:281–287.

Rouphael, A. B., and G. J. Inglis. 2002. Increased spatial and temporal variability in coral damage caused by recreational scuba diving. Ecological Applications **12**:427–440.

Sanecki, G. M., K. Green, H. Wood, and D. Lindenmayer. 2006. The implications of snow-based recreation for small mammals in the subnivean space in south-east Australia. Biological Conservation **129**:511–518.

Sarmento VC, Barreto AF, S, Santos PJ, P. 2013. Recovery of meiofauna following a short-term disturbance on coral reefs. Biodiversity & Conservation **22**:2645–2663.

Schierding, M., S. Vahder, L. Dau, and U. Irmler. 2011. Impacts on biodiversity at Baltic Sea beaches. Biodiversity and Conservation **20**:1973–1985.

Schlacher, T. A., and L. Thompson. 2012. Beach recreation impacts benthic invertebrates on ocean-exposed sandy shores. Biological Conservation **147**:123–132.

Schummer, M. L., and W. R. Eddleman. 2003. Effects of disturbance on activity and energy budgets of migrating waterbirds in south-central Oklahoma. Journal of Wildlife Management **67**:789–795.

Schurr, M., A. Fuentes, E. Luecke, J. Cortes, and E. Shaw. 2012. Intergroup variation in stable isotope ratios reflects anthropogenic impact on the Barbary macaques (*Macaca sylvanus*) of Gibraltar. Primates **53**:31–40.

Sebastianutto, L., M. Picciulin, M. Costantini, and E. A. Ferrero. 2011. How boat noise affects an ecologically crucial behaviour: the case of territoriality in *Gobius cruentatus* (Gobiidae). Environmental Biology of Fishes **92**:207–215.

Seip, D. R., C. J. Johnson, and G. S. Watts. 2007. Displacement of mountain caribou from winter habitat by snowmobiles. Journal of Wildlife Management **71**:1539–1544.

Selman W, Qualls C, Owen JC. 2013. Effects of human disturbance on the behavior and physiology of an imperiled freshwater turtle. The Journal of Wildlife Management **77**:877–885.

Semeniuk, C. A. D., S. Bourgeon, S. L. Smith, and K. D. Rothley. 2009. Hematological differences between stingrays at tourist and non-visited sites suggest physiological costs of wildlife tourism. Biological Conservation **142**:1818–1829.

Semeniuk, C. A. D., and K. D. Rothley. 2008. Costs of group-living for a normally solitary forager: effects of provisioning tourism on southern stingrays *Dasyatis americana*. Marine Ecology Progress Series **357**:271–282.

Semenova, O. V. 2008. Ecology of ground beetles in an industrial city. Russian Journal of Ecology **39**:444–450.

Sheppard, N., K. A. Pitt, and T. A. Schlacher. 2009. Sub-lethal effects of off-road vehicles (ORVs) on surf clams on sandy beaches. Journal of Experimental Marine Biology and Ecology **380**:113–118.

Shively, K. J., A. W. Alldredge, and G. E. Phillips. 2005. Elk reproductive response to removal of calving season disturbance by humans. Journal of Wildlife Management **69**:1073–1080.

Shutt K et al. 2014. Effects of habituation, research and ecotourism on faecal glucocorticoid metabolites in wild western lowland gorillas: Implications for conservation management. Biological Conservation **172**:72–79.

Sibbald, A. M., R. J. Hooper, J. E. McLeod, and I. J. Gordon. August. Responses of red deer (*Cervus elaphus*) to regular disturbance by hill walkers. European Journal of Wildlife Research **57**:817–825.

Skagen, S. K., R. L. Knight, and G. H. Orians. 1991. Human disturbance of an avian scavenging guild. Ecological Applications **1**:215–225.

Skagen, S. K., C. P. Melcher, and E. Muths. 2001. The interplay of habitat change, human disturbance and species interactions in a waterbird colony. American Midland Naturalist **145**:18–28.

Smith-Castro, J. R., and A. D. Rodewald. 2010. Effects of Recreational Trails on Northern Cardinals (Cardinalis cardinalis) in Forested Urban Parks. Natural Areas Journal **30**:328–337.

St Clair, J. J. H., G. E. García-Peña, R. W. Woods, and T. Székely. 2010. Presence of mammalian predators decreases tolerance to human disturbance in a breeding shorebird. Behavioral Ecology **21**:1285–1292.

Stalmaster, M. V., and J. L. Kaiser. 1998. Effects of recreational activity on wintering bald eagles. Wildlife Monographs:3–46.

Stamation, K. A., D. B. Croft, P. D. Shaughnessy, K. A. Waples, and S. V. Briggs. 2010. Behavioral responses of humpback whales (*Megaptera novaeangliae*) to whale-watching vessels on the southeastern coast of Australia. Marine Mammal Science **26**:98–122.

Steckenreuter, A., R. Harcourt, and L. Moeller. 2011. Distance does matter: close approaches by boats impede feeding and resting behaviour of Indo-Pacific bottlenose dolphins. Wildlife Research **38**:455–463.

Steidl, R. J., and R. G. Anthony. 1996. Responses of bald eagles to human activity during the summer in interior Alaska. Ecological Applications **6**:482–491.

Steidl, R. J., and R. G. Anthony. 2000. Experimental effects of human activity on breeding bald eagles. Ecological Applications **10**:258–268.

Steiner, A. J., and S. P. Leatherman. 1981. Recreational impacts on the distribution of ghost crabs *Ocypode quadrata* fab. Biological Conservation **20**:111–122.

Stensland, E., and P. Berggren. 2007. Behavioural changes in female Indo-Pacific bottlenose dolphins in response to boat-based tourism. Marine Ecology Progress Series **332**:225–234.

St-Louis A, Hamel S, Mainguy J, Côté SD. 2013. Factors influencing the reaction of mountain goats towards all-terrain vehicles. The Journal of Wildlife Management **77**:599–605.

Stockin, K. A., D. Lusseau, V. Binedell, N. Wiseman, and M. B. Orams. 2008. Tourism affects the behavioural budget of the common dolphin *Delphinus sp*. in the Hauraki Gulf, New Zealand. Marine Ecology Progress Series **355**:287–295.

Storch, I., and C. Leidenberger. 2003. Tourism, mountain huts and distribution of corvids in the Bavarian Alps, Germany. Wildlife Biology **9**:301–308.

Swarthout, E. C. H., and R. J. Steidl. 2003. Experimental effects of hiking on breeding Mexican spotted owls. Conservation Biology **17**:307–315.

Tadesse, S. A., and B. P. Kotler. 2012. Impact of tourism on Nubian Ibex (*Capra nubiana*) revealed through assessment of behavioral indicators. Behavioral Ecology **23**:1257-1262.

Tarjuelo R, Barja I, Morales MB, Traba J, Benítez-López A, Casas F, Arroyo B, Delgado MP, Mougeot F. 2015. Effects of human activity on physiological and behavioral responses of an endangered steppe bird. Behavioral Ecology **26**:828–838.

Taylor, A. R., and R. L. Knight. 2003. Wildlife responses to recreation and associated visitor perceptions. Ecological Applications **13**:951–963.

Taylor DP, Vradenburg JN, Smith LM, Lovern MB, McMurry ST. 2014. Effects of anthropogenic and environmental stress on the corticosterone levels of wintering Northern Pintails ( Anas acuta). Canadian Journal of Zoology **92**:185–193.

Thiel, D., S. Jenni-Eiermann, V. Braunisch, R. Palme, and L. Jenni. 2008. Ski tourism affects habitat use and evokes a physiological stress response in capercaillie *Tetrao urogallus*: a new methodological approach. Journal of Applied Ecology **45**:845–853.

Thiel, D., E. Menoni, J.-F. Brenot, and L. Jenni. 2007. Effects of recreation and hunting on flushing distance of capercaillie. Journal of Wildlife Management **71**:1784–1792.

Thomas, K., R. G. Kvitek, and C. Bretz. 2003. Effects of human activity on the foraging behavior of sanderlings *Calidris alba*. Biological Conservation **109**:67–71.

Titus, J. R., and L. W. VanDruff. 1981. Response of the common loon to recreational pressure in the Boundary Waters Canoe Area, northeastern Minnesota. Wildlife Monographs:3–59.

Tratalos, J. A., and T. J. Austin. 2001. Impacts of recreational SCUBA diving on coral communities of the Caribbean island of Grand Cayman. Biological Conservation **102**:67–75.

Trathan, P. N., J. Forcada, R. Atkinson, R. H. Downie, and J. R. Shears. 2008. Population assessments of gentoo penguins (*Pygoscelis papua*) breeding at an important Antarctic tourist site, Goudier Island, Port Lockroy, Palmer Archipelago, Antarctica. Biological Conservation **141**:3019–3028.

Trulio, L. A., and J. Sokale. 2008. Foraging shorebird response to trail use around San Francisco Bay. Journal of Wildlife Management **72**:1775–1780.

Tsounis, G., L. Martinez, L. Bramanti, N. Viladrich, J. Gili, A. Martinez, and S. Rossi. 2012. Anthropogenic effects on reproductive effort and allocation of energy reserves in the Mediterranean octocoral *Paramuricea clavata*. Marine Ecology Progress Series **449**:161–172.

Tull, J. C., and P. F. Brussard. 2007. Fluctuating asymmetry as an indicator of environmental stress from off-highway vehicles. Journal of Wildlife Management **71**:1944–1948.

Ulrich, W., M. Zalewski, I. Hajdamowicz, M. Stańska, W. Ciurzycki, and P. Tykarski. 2010. Tourism disassembles patterns of co-occurrence and weakens responses to environmental conditions of spider communities on small lake islands. Community Ecology **11**:5–12.

Uyarra, M. C., and I. M. Côté. 2007. The quest for cryptic creatures: impacts of species-focused recreational diving on corals. Biological Conservation **136**:77–84.

Van der Zande, A. N., J. C. Berkhuizen, H. C. van Latesteijn, W. J. ter Keurs, and A. J. Poppelaars. 1984. Impact of outdoor recreation on the density of a number of breeding bird species in woods adjacent to urban residential areas. Biological Conservation **30**:1–39.

Van der Zande, A. N., and P. Vos. 1984. Impact of a semi-experimental increase in recreation intensity on the densities of birds in groves and hedges on a lake shore in The Netherlands. Biological Conservation **30**:237–259.

van Riper CI, Fontaine JJ, Wagtendonk JW van. 2013. Great Gray Owls (Strix nebulosa) in Yosemite National Park: On the Importance of Food, Forest Structure, and Human Disturbance. Natural Areas Journal **33**:286–295.

Velando, A., and I. Munilla. 2011. Disturbance to a foraging seabird by sea-based tourism: Implications for reserve management in marine protected areas. Biological Conservation **144**:1167–1174.

Veloso, V. G., E. S. Silva, C. H. S. Caetano, and R. S. Cardoso. 2006. Comparison between the macroinfauna of urbanized and protected beaches in Rio de Janeiro State, Brazil. Biological Conservation **127**:510–515.

Villanueva C, Walker BG, Bertellotti M. 2014. Seasonal variation in the physiological and behavioral responses to tourist visitation in Magellanic penguins. The Journal of Wildlife Management **78**:1466–1476.

Walker, B. G., P. D. Boersma, and J. C. Wingfield. 2005. Physiological and behavioral differences in Magellanic penguin chicks in undisturbed and tourist-visited locations of a colony. Conservation Biology **19**:1571–1577.

Walker, B. G., P. Dee Boersma, and J. C. Wingfield. 2006. Habituation of adult magellanic penguins to human visitation as expressed through behavior and corticosterone secretion. Conservation Biology **20**:146–154.

Wang Y, Allen ML, Wilmers CC. 2015. Mesopredator spatial and temporal responses to large predators and human development in the Santa Cruz Mountains of California. Biological Conservation **190**:23–33.

Watson H, Bolton M, Monaghan P. 2014. Out of sight but not out of harm’s way: Human disturbance reduces reproductive success of a cavity-nesting seabird. Biological Conservation **174**:127–133.

Weinrich, M., and C. Corbelli. 2009. Does whale watching in Southern New England impact humpback whale (*Megaptera novaeangliae*) calf production or calf survival? Biological Conservation **142**:2931–2940.

Williams, R., and E. Ashe. 2007. Killer whale evasive tactics vary with boat number. Journal of Zoology **272**:390–397.

Yalden, D. W. 1992. The influence of recreational disturbance on common sandpipers *Actitis hypoleucos* breeding by an upland reservoir, in England. Biological Conservation **61**:41–49.

Yalden, D. W. 1990. Recreational disturbance of large mammals in the Peak District. Journal of Zoology **221**:293–298.

Yalden, P. E., and D. W. Yalden. 1990. Recreational disturbances of breeding golden plovers *Pluvialis apricarius*. Biological Conservation **51**:243–262.

Yasué, M., and P. Dearden. 2006. The potential impact of tourism development on habitat availability and productivity of Malaysian plovers *Charadrius peronii*. Journal of Applied Ecology **43**:978–989.

Zakai, D., and N. E. Chadwick-Furman. 2002. Impacts of intensive recreational diving on reef corals at Eilat, northern Red Sea. Biological Conservation **105**:179–187.

Zhang M, Wang X, Ding Y. 2013. Flight responses of blue sheep in Ningxia Helan Mountain National Nature Reserve. Folia Zoologica **62**:185–192.

Zhou Y, Buesching CD, Newman C, Kaneko Y, Xie Z, Macdonald DW. 2013. Balancing the benefits of ecotourism and development: The effects of visitor trail-use on mammals in a Protected Area in rapidly developing China. Biological Conservation **165**:18–24.

Zielinski, W. J., K. M. Slauson, and A. E. Bowles. 2008. Effects of off-highway vehicle use on the American marten. Journal of Wildlife Management **72**:1558–1571.

Zolotarev MP, Belskaya EA. 2015. Ground-dwelling invertebrates in a large industrial city: Differentiation of recreation and urbanization effects. Contemporary Problems of Ecology **8**:83–90.

Zuberogoitia, I., J. Zabala, J. A. Martínez, J. E. Martínez, and A. Azkona. 2008. Effect of human activities on Egyptian vulture breeding success. Animal Conservation **11**:313–320.

Zwijacz-Kozica T, Selva N, Barja I, Silván G, Martínez-Fernández L, Illera JC, Jodłowski M. 2012. Concentration of fecal cortisol metabolites in chamois in relation to tourist pressure in Tatra National Park (South Poland). Acta Theriologica **58**:215–222.
